# In Silico Screening of the DrugBank Database to Search for Possible Drugs against SARS-CoV-2

**DOI:** 10.3390/molecules26041100

**Published:** 2021-02-19

**Authors:** Sebastián A. Cuesta, José R. Mora, Edgar A. Márquez

**Affiliations:** 1 Grupo de Química Computacional y Teórica (QCT-USFQ), Departamento de Ingeniería Química, Colegio Politécnico, Universidad San Francisco de Quito, Diego de Robles y Vía Interoceánica, Quito 170901, Ecuador; sebascuestahoyos89@gmail.com; 2 Grupo de Investigaciones en Química y Biología, Departamento de Química y Biología, Facultad de Ciencias Exactas, Universidad del Norte, Carrera 51B, Km 5, vía Puerto Colombia, Barranquilla 081007, Colombia

**Keywords:** SARS-CoV-2, QSAR, docking analysis, DrugBank, molecular dynamics

## Abstract

Coronavirus desease 2019 (COVID-19) is responsible for more than 1.80 M deaths worldwide. A Quantitative Structure-Activity Relationships (QSAR) model is developed based on experimental pIC_50_ values reported for a structurally diverse dataset. A robust model with only five descriptors is found, with values of R^2^ = 0.897, Q^2^_LOO_ = 0.854, and Q^2^_ext_ = 0.876 and complying with all the parameters established in the validation Tropsha’s test. The analysis of the applicability domain (AD) reveals coverage of about 90% for the external test set. Docking and molecular dynamic analysis are performed on the three most relevant biological targets for SARS-CoV-2: main protease, papain-like protease, and RNA-dependent RNA polymerase. A screening of the DrugBank database is executed, predicting the pIC_50_ value of 6664 drugs, which are IN the AD of the model (coverage = 79%). Fifty-seven possible potent anti-COVID-19 candidates with pIC_50_ values > 6.6 are identified, and based on a pharmacophore modelling analysis, four compounds of this set can be suggested as potent candidates to be potential inhibitors of SARS-CoV-2. Finally, the biological activity of the compounds was related to the frontier molecular orbitals shapes.

## 1. Introduction

At the end of December 2019, China reported to the WHO several cases of human respiratory disease in Wuhan, Hubei Province. A novel, highly pathogenic coronavirus strain (CoV) was linked to this fatal pneumonia called COVID-19 [1,2,3]. By January 30, The Public Health Emergency of International Concern (PHEIC) was announced for this new CoV outbreak [2]. The disease expanded dramatically worldwide, being recognized by the WHO as a pandemic in mid-March [1,2,4]. This virus, related to the severe acute respiratory syndrome (SARS) that appeared in 2002 and the Middle East respiratory syndrome-related coronavirus (MERS) of 2012, was named SARS-CoV-2 [1,5].

Biochemically, CoVs are enveloped, positive-sense, single-stranded RNA viruses classified as α, β, γ, and δ [1,2,3,6]. CoV infects humans as well as other animals such as bats, camels, pigs, and pangolins [7]. It was discovered in the 1960s and was thought to cause only a mild disease [5], but SARS-CoV-2 is the third emergence of a coronavirus in less than 20 years with important mortality rates. In this sense, SARS, MERS, and SARS-CoV-2 have death rates of 9.6%, 35.5% and 6.76%, respectively [8,9]. Early COVID-19 symptoms include fatigue, dry cough, muscle soreness, shortness of breath, and fever [10]. In severe cases of infections with SARS-CoV-2, patients require endotracheal intubation and mechanical ventilation, as they develop acute respiratory distress syndrome, pulmonary oedema, and severe pneumonia, causing death from sepsis shock, respiratory, or multiple organ failures [8,11]. 

SARS-CoV-2 spread has not stopped, being in more than 215 countries, infecting more than 25 million people, and causing more than 1.8 million fatalities, up to December 2020 [1,12,13,14,15,16].

The fast spread of the virus has caused unwarranted fatalities, and the collapse of health-care systems and the economy worldwide [15,17]. Currently, there is no effective treatment available for CoV infections, including COVID-19 [14]. Therefore, efforts to create new therapies, such as antiviral medication or vaccines, are vital to reduce mortality, reactivate the economy, and return to a normal lifestyle [4,8,12,17].

It is well known that a drug discovery and development process takes a long time and has high costs. Therefore, a different approach is needed to find a solution in the short term [4,15]. Having the RNA genome sequence is an important starting point to develop an effective treatment [17]. Testing existing broad-spectrum antiviral agents through experimental as well as theoretical and machine learning methods is one of the most used approaches [1,18,19,20]. Additionally, studying drugs that were developed for a different purpose (drug repurposing) to treat COVID-19 has emerged as a successful strategy due to reduced cost, clinical risk, and time [12,13,15,20].

Antiviral drugs can either target the virus directly through the inhibition of essential enzymes or indirectly by host cell modulation [7,15]. CoVs present two main proteases, the papain-like protease (PLpro) and the 3-chymotrypsin-like protease, also called main protease (Mpro), which are the ones in charge of cleaving the 800 kDa polypeptide produced from genome transcription [1,2,4,15]. Mpro and PLpro produce many non-structural proteins (NSPs), including Helicase and RNA-dependent RNA polymerase (RdRp) [1,2,7,17]. These proteases are essential for viral replication and transcription, which make them very interesting targets for an effective drug discovery approach [1,7]. Another interesting target for its importance in the RNA genome replication and protein synthesis is RdRp [17]. These three enzymes are highly conserved between SARS-CoV-2 and SARS-CoV. RdRp even presents a conserved active site between different RNA viruses, including the hepatitis virus [5,6,14,21].

Currently, several drugs have been under clinical trials against COVID-19. The Solidarity Trial of the WHO announced, at the end of 2020, that treatments with hydroxychloroquine, remdesivir, interferon and lopinavir/ritonavir showed no effect in patients’ recovery, hospital stay, artificial ventilation, and mortality [22,23]. Furthermore, clinical trials performed using baloxavir, marboxil and favipiravir did not show improvements during SARS-CoV-2 infection [24]. Therefore, efficient treatment has not been found yet.

In the literature, several studies have been carried out trying to find effective ways to fight COVID-19. These include in vitro studies with old drugs (ivermectin) [25], nanotechnology-based strategies [16], antimicrobial peptides such as Lactoferrin [26], target RdRp with a series of nucleotide analogues [14,27], transcriptomic and proteomic approaches [13], fragment molecular orbital based interaction analyses [4], the screening of medicinal plants’ active compounds [2] and Food and Drug Administration (FDA) approved drugs [21] against Mpro, molecular mechanics/Poisson−Boltzmann surface area/weighted solvent-accessible surface area analysis [20], S-protein—ACE2 interface docking through homology modelling [11,18], etc. From these studies, many chemical compounds, including antiviral agents, vitamins, and antibiotics, have emerged as possible drugs to treat COVID-19. Still, a robust computational model based on experimental data has not been published yet. In this sense, this work presents a predictive Quantitative Structure-Activity Relationships (QSAR) model, based on the recently reported experimental pIC_50_ values for a series of molecules selected from the FDA approved database [28]. Furthermore, an exhaustive docking analysis was done, taking into account three possible targets, considered relevant in COVID-19 treatment: SARS-CoV-2 Mpro, PLpro, and RdRp. Finally, a screening of the DrugBank 5.1.7 [29] database was carried out to find other possible drugs against SARS-CoV-2.

## 2. Results and Discussion

### 2.1. Data Set

**Figure 1:**
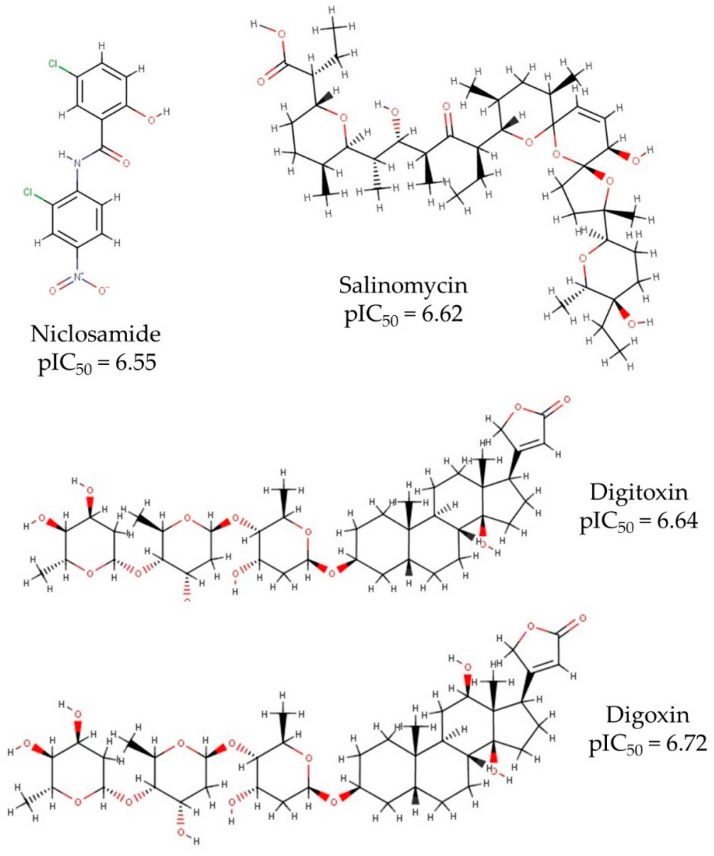
Four most potent antiviral candidates, based on experimental pIC_50_ values.

The dataset reported by Jeon et al. [28] was used as the initial input in the present study. This dataset lists experimental IC_50_ values of different drugs, being the largest and most diverse dataset reported, so far, for the treatment of COVID-19 using experimental techniques. The molecules tested in this dataset were extracted from the FDA approved drugs, suggesting a safe alternative as potent antiviral candidates against SARS-CoV-2. Initially, the 2D structures of these compounds were extracted from the PubChem webpage (https://pubchem.ncbi.nlm.nih.gov/ (accessed on 18 February 2021)) and optimized at the Universal Force Field (UFF) level of theory to obtain the 3D structures. In the Supplementary Materials (SM1 and SM2), 2D structures (.sdf) for all the datasets are available. Figure 1 shows the four most potent candidates: niclosamide, salinomycin, digitoxin, and digoxin, with pIC_50_ > 6.4. The most active compound is digoxin, which is used in the treatment of some heart diseases, such as atrial fibrillation, atrial flutter, and heart failure [30]. Digoxin is categorized as a positive inotropic and negative chronotropic drug, which implies that it increases the force of the heartbeat but decreases the heart rate. Digitoxin is a digoxin analogous compound with the only difference being an OH group, present in digoxin, but missing in digitoxin (Figure 1). Digitoxin is also a cardiac glycoside, which is commonly used instead of digoxin, mainly in patients with erratic kidney function, as digitoxin has hepatic elimination, whereas digoxin proceeds through renal excretion [31]. Salinomycin is often employed as a useful anti-bacterial and antibiotic agent [32], being administrated for the treatment of coccidiosis [33]. Niclosamide is reported as an anthelminthic and antineoplastic drug, used against tapeworm infections [34]. Based on safe alternatives, niclosamide was suggested as the best candidate to be a drug for the treatment of COVID-19.

**Figure 2:**
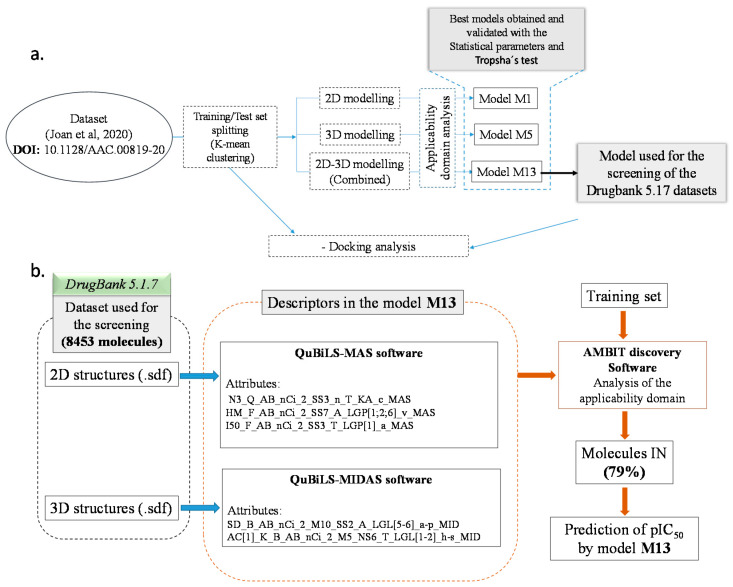
(**a**) Quantitative Structure-Activity Relationships (QSAR) and molecular docking simulation approach to finding possible drugs against SARS-CoV-2. (**b**) DrugBank screening and pIC_50_ prediction procedure.

For the QSAR analysis, pIC_50_ = −log(IC_50_,M) experimental values were employed as the response variable, and three different modelling processes were performed (Figure 2a). In the first one, 2D molecular descriptors were obtained by employing QuBiLS-MAS indexes; called “2D modelling” from now on. The second simulation was performed using the 3D optimized structures to produce 3D molecular descriptors employing the QuBiLS-MIDAS indexes; this is further cited as “3D modelling”. The third modelling process was a combination of 2D and 3D descriptors—“2D-3D modelling”.

### 2.2. Dataset Splitting (Training/Test)

**Figure 3:**
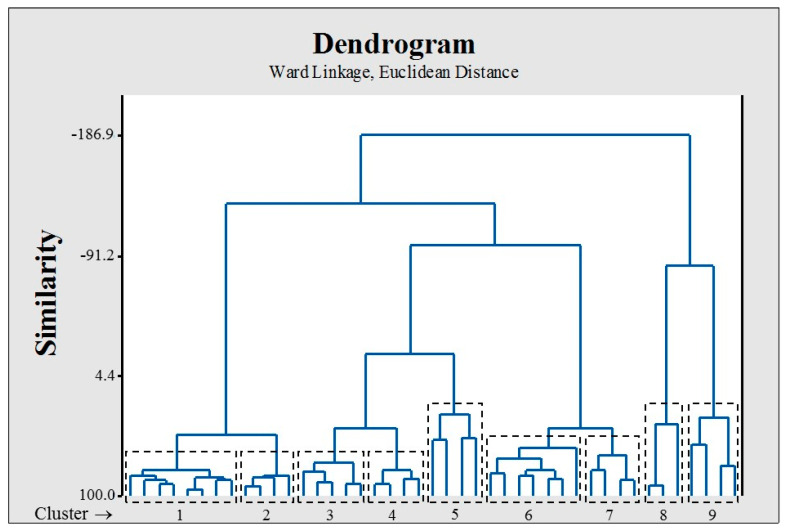
Dendrogram obtained for the graphical representation of the nine clusters used for the training/test set splitting.

After selecting the attributes, a rational separation of the dataset into training (35 compounds) and test set (9 compounds) was performed, employing a K-means cluster analysis, taking into account the separation of the dataset in nine clusters, and selecting ~20% of each cluster as a test set (Figure 3).

The Euclidean distances were employed as the similarity metric in different instances. The applicability domain (AD) of the training set was evaluated by employing the four different methods implemented in AMBIT discovery [35], i.e., principal component analysis (PCA) range, Euclidean distance, citi-block distance, and probability density. The consensus approach was applied as implemented in Ambit discovery, i.e., if a molecule is labelled as out by two or more methods, then the molecule is considered as OUT. Only one molecule of the test set (ebastine) was found to be OUT the AD for the four employed methods, resulting in 90% coverage.

### 2.3. QSAR Modelling

In the first stage of model construction, it is mandatory to evaluate the co-linearity between the variables, which is normally achieved by obtaining the correlation matrix for the models. In this study, all models containing some attributes between 4 and 7 were selected, reaching 13 models. From these models, the most robust for the 2D, 3D, and 2D-3D modellings were chosen (M1, M5 and M13, respectively). Pearson’s correlation coefficients between an attribute and the rest are shown graphically in Figure S1 (SM3). As it can be observed, the bar graph for each descriptor shows the minimum (on the left) and the maximum (on the right) value for the Pearson’s coefficients correlation of the descriptor with the others. Therefore, the most negative value is −0.45, and the most positive value is 0.60, showing no co-linearity between the attributes in the three models.

Regarding the molecular attributes, the names are related to the mathematical approaches employed for the calculation of these topographic indexes, which are weighed by different physicochemical properties, denoted by the lowercase letters at the end of each descriptor. The physicochemical properties involved in model M1, M5, and M13 are electronegativity (e), softness (s), hardness (h), Van der Waals volume (v), charges (c), polarizability (p), polar surface area (PSA), refractivity index (r), and AlogP (a). Hardness (h) and softness (s) give information about the donor-acceptor properties of a molecule [36]. The PSA is related to the hydrophilic interaction and the formation of hydrogen bonds [37]. The electronegativity (e) is related to the existence of heteroatoms, involving electrostatic protein-ligand interactions [38,39,40]. The polarizability and the refractive index are associated with short-range interaction between the drug and the active site of the protein [41]. The AlogP is the partition coefficient octanol/water, which provides information about the solubility of the molecule in polar and non-polar systems.

**Figure 4:**
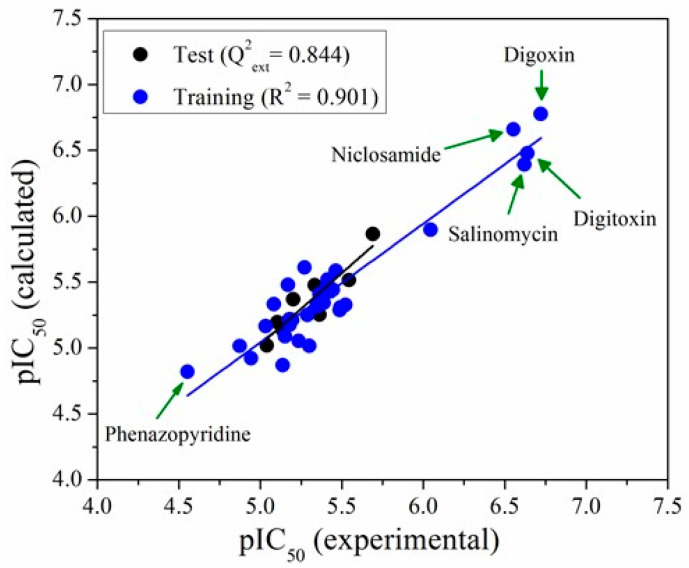
Experimental vs. calculated pIC_50_ values by the model M13.

Model M13, with only five descriptors, was selected as the most robust. It was constructed by employing multiple linear regression techniques, and the standardized coefficients by multiplying each standardized variable involved in the mathematical representation of each case, as shown in Equation (1). Models 1–12 are given in the Supplementary Material (Table S1, SM3). In Figure 4, a graphical representation of experimental data vs. predictions of model M13 is presented. It can be seen that there is a good fitting between calculated and experimental pIC_50_ values in either the training or test sets (Equation (1)).

pIC_50_ = 4.5482 + 0.4998A^2D-3D^ − 0.3296B^2D-3D^ + 0.3120C^2D-3D^ − 0.8384D^2D-3D^ + 0.8265E^2D-3D^ (1)

Table 1 shows all the statistical parameters used for M13 validation. The coefficient of determination (R^2^) close to one, Fisher coefficient (F) values greater than 30 and small residual standard deviation (s) values suggest a good fitting. The values of the cross-validation coefficients for the leave-one-out (Q2LOO) and the external validation (Q2ext) are bigger than 0.8, indicating that these models are robust enough for the prediction of pIC_50_. The Y-scrambling analysis shows that there is no correlation by chance, as demonstrated by the small values obtained for a(R2) and a(Q2). The Organization for Economic Cooperation and Development (OECD) principles [42] establish Tropsha’s test as an exhaustive validation procedure determining if a model is robust for the predictability of a given response variable. In this sense, Tropsha’s test was applied to the three models. PASS values for all the models prove the good performance in the predictability of the external test set as well as in the leave-one-out cross-validation.

### 2.4. DrugBank 5.1.7 Screening

The screening procedure is depicted in Figure 2b. Model M13 was employed for the screening of four important datasets reported in DrugBank: approved, experimental, nutraceutical, and withdrawn. Then, the datasets were cleaned by removing inorganic, organometallic, salts, and mixture compounds, obtaining 2265 molecules in the approved dataset, 5858 in the experimental, 102 in the nutraceutical, and 228 in the withdrawn drugs dataset (Table 2). After that, the AD of these datasets was evaluated into the model M13 training set, finding that 79% of the drugs are IN the AD. This good coverage demonstrates the robustness of the model and the reliability of the prediction, fact related to the molecular diversity found in the training dataset. The pIC_50_ values of these compounds were estimated and are presented in the Supplementary Material (SM4). The intervals of the predicted pIC_50_ values for each dataset are presented in Table 2.

From the total dataset evaluated in this screening, it is possible to identify other possible potent antiviral candidates, which could be used for the treatment of COVID-19. In this respect, 57 compounds were found to have predicted pIC_50_ values > 6.6. The two best compounds (DB08476 and DB07287) present much higher pIC_50_ values (7.5) compared to the other ones (≤7.1). These compounds, 3-amino-azacyclotridecan-2-one (DB08476) and 2-(2,4-dichlorophenoxy)-5-(pyridin-2-ylmethyl)phenol (DB07287), are experimental drugs belonging to the macrolactam and diphenylethers classes, respectively [29]. Interestingly, these types of compounds are used as antibiotic drugs [43,44,45,46].

Regarding the 57 compounds with pIC_50_ values > 6.6, miconazole (DB01110) is an azole class antifungal used to treat pityriasis Versicolor, ringworm, and infections caused by *Candida* sp. [47,48]. Nitenpyram (DB11438) is a nicotinic acetylcholine receptor inhibitor. It is used to treat *Ctenocephalides* spp. in dogs and cats and is rapidly eliminated in urine. Furthermore, nitenpyram is also considered a second-generation pesticide of the neonicotinoid family [49]. Metildigoxin (DB13401) is a semi-synthetic cardiac glycoside prodrug prescribed to treat arrhythmia and heart failure [50]. After oral administration, it is completely absorbed and rapidly transformed into digoxin [51]. Chemically, it is closely related to digoxin, changing a hydroxyl group in the latter for a methoxy one on the terminal monosaccharide [52]. Furthermore, 2′,4′-Dinitrophenyl-2deoxy-2-Fluro-B-D-Cellobioside (DB04086) is an experimental drug belonging to the class of o-glycosyl organic compounds [53].

Interestingly, some of the commonly used drugs to treat respiratory problems, bronchitis, asthma, and allergic rhinitis were also identified as possible good candidates against SARS-CoV-2. These include dirithromycin (DB00954), a macrolide glycopeptide antibiotic used to treat upper and lower respiratory infections [54,55,56], monensin (DB11430), flunisolide (DB00180), fluticasone propionate (DB00588), and tixocortol (DB09091). In addition, some antibiotics, widely used in many infections, were identified as potent SARS-CoV-2 inhibitors, such as amikacin, streptomycin, lincomycin, and spiramycin. The latter is used for the treatment of toxoplasmosis in pregnant women.

Recently, a drug database against SARS-CoV-2 called DockCov2 was published [57]. The results of the experimental pIC_50_ obtained from the Jeon et al. [28] database were compared to DockCov2 scores for RdRp, Mpro, and the highest score obtained for each compound independently of the target (Table S2, SM3). From the 44 molecules, only 27 were present in the DockCov2 database. The results showed a poor correlation (R^2^ < 0.11) between experimental and DockCoV2 values, failing to predict the affinity of the Jeon et al. [28] database.

The strength that makes M13 a robust pIC_50_ prediction model is the use of experimental data and that a biological target does not need to be identified. The methods reported, such as the ones using a double (Autodock Vina and MM-GBSA) scoring approach [58] and virtual screening [59], rely only on in silico methodology, with the flaws these methods have including the requirement of a specific biological target.

**Figure 5:**
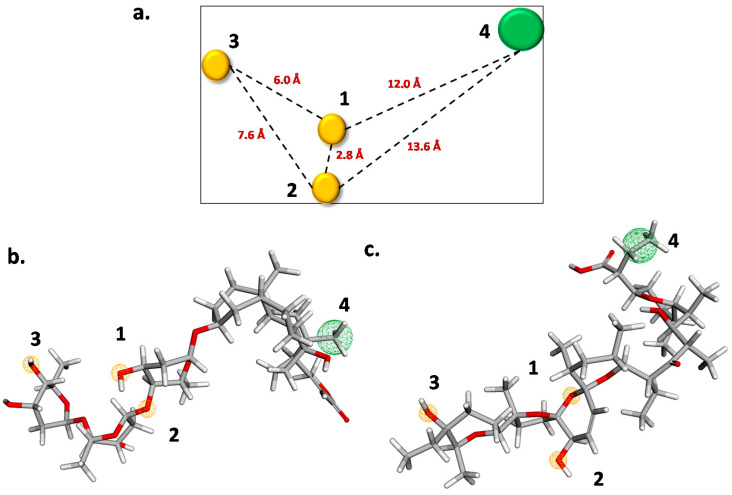
(**a**) Pharmacophore result for the four most active structures. Pharmacophore overlaid to digoxin (**b**) and salinomycin (**c**) Hydrogen Bond acceptors are presented in yellow and hydrophobic interactions in green.

This screening was complemented with pharmacophore modelling. For the model construction, the four most active compounds extracted from the Jeon et al. [28] database were used (digoxin, digitoxin, salinomycin, and niclosamide). The results obtained from the Pharmagist web server (https://bioinfo3d.cs.tau.ac.il/PharmaGist/ (accessed on 18 February 2021)) show that the pharmacophore is composed of three hydrogen bond acceptors (HBA) and one hydrophobic (HPH) interaction (Figure 5). The four features and a combination of three features were scanned against the experimental database and tabulated in Table 3.

Looking at the number of hits obtained, models 4 and 5 (Table 3) were chosen as the best ones and used for DrugBank screening. Model 4 is composed of two HBAs and one HPH interaction. The distance between the two HBAs is 2.8 Å, while the distance from HBA1/HBA2 to HPH4 is 12.0 Å and 13.6 Å, respectively. For model 5, the pharmacophore is formed by three HBAs with a distance of 2.8 Å between HBA1 and HBA2, 6.0 Å between HBA1 and HBA3, and 7.6 Å between HBA2 and HBA3. Model 4 and model 5 were tested against the Jeon et al. [28] database (Table S3, SM3), where 15 compounds matched model 4 with RMSD values lower than 0.54. In model 5, 14 compounds matched the pharmacophore with RMSD values lower than 0.32. This is not surprising as QSAR model M13 is based on experimental data and is not based on a single biological target.

DrugBank screening produced 48 hits for model 4 and 45 hits for model 5. As the same compound can produce several hits by changing its pose, the lists were cleaned, leaving the pose for each compound with the lowest RMSD. Then, the pIC_50_ value of each hit was predicted and filtered (pIC_50_ > 6.6). Six compounds were obtained for model 4 and seven for model 5 (Table S4, SM3). Three compounds, DB01980, DB03259, and DB02438, fit both models. DB03259 was compared to the pharmacophore model 4, finding a distance between both HBAs of 3.1 Å, between HBA1 and HPH4 of 5.6 Å, and between HBA2 and HPH4 of 7.1 Å (Figure S2a, SM3). The same was performed with DB02438 and pharmacophore model 5, finding a better fit to the model with a difference in the HBA1–HBA2 distance of 0.0 Å, HBA1–HBA3 of 0.5 Å, and HBA2–HBA3 of 0.1 Å (Figure S2b, SM3).

### 2.5. Docking Study

As described in the methodology section, molecules taken from the Jeon et al. [28] database were docked against Mpro, PLpro, RdRp, and tabulated in Table S5. Furthermore, the two molecules with the biggest predicted pIC_50_ value, plus the best two compounds obtained from the pharmacophore model 4 and the best two from the pharmacophore model 5, were also considered, and their results are presented in Table S6 (six compounds total). The calculation area was reduced to the active site spotted experimentally. When knowing the active site, reducing the calculation area to it is an efficient approach, as all the computational cost is used in finding the right pose, rather than looking for the active site in the entire enzyme. Ligands extracted from the experimental structure were also docked as a validation procedure, finding that, for Mpro and PLpro, the docked structure superimposed the experimental one. For RdRp, experimental and docked structures are in different places because its Cryo-EM structure was elucidated when remdesivir was already incorporated to the RNA strand and outside the active site.

Docking scores range from −4.7 to −9.2 kcal/mol in Mpro, from −4.7 to −8.5 kcal/mol in PLpro, and from −4.5 to −9.4 kcal/mol in RdRp, showing that all molecules in the dataset might be able to fit in any of the three enzymes active sites inhibiting them, and affecting the SARS-CoV-2 life cycle. Interestingly, the highest docking scores were found for digoxin, which is the molecule with the biggest pIC_50_ (6.72). Digitoxin and Salinomycin, which present the second and third highest pIC_50_ values, also present higher docking scores, being −7.8 and −8.0 kcal/mol for MP, −8.9 and −7.2 kcal/mol for PL, and −9.3 and −8.6 kcal/mol for RdRp, respectively. Docking affinity scores were compared with experimental pIC_50_ values, finding a poor correlation with r^2^ values below 0.17. It is well-known that docking scores are not good for predicting binding affinities [60,61]. Most docking scoring functions depend on the size of the ligand, as large molecules will have more functional groups and make more interactions with the active site. Still, docking scores are very useful to separate which molecules can bind to a biological target and which ones are less likely or definitely will not fit in an enzymatic pocket. Furthermore, docking is key to study the different interactions and see how changes in the molecule can affect how it fits the active site. A deep analysis of the different poses and interactions with the enzyme allows us to filter the best candidates. In this sense, seven drugs were chosen from the dataset—four from the top of the list and three from the bottom. The dataset was ordered from highest to lowest pIC_50_ values, and digoxin, hexachlorophene, bazedoxifene, dronedarone, thioridazine, chloroquine, and remdesivir were selected. Moreover, the six molecules chosen from the DrugBank screening were also considered. Table 4 shows the different interactions resulting from the docking calculation of these thirteen drugs against Mpro.

**Figure 6:**
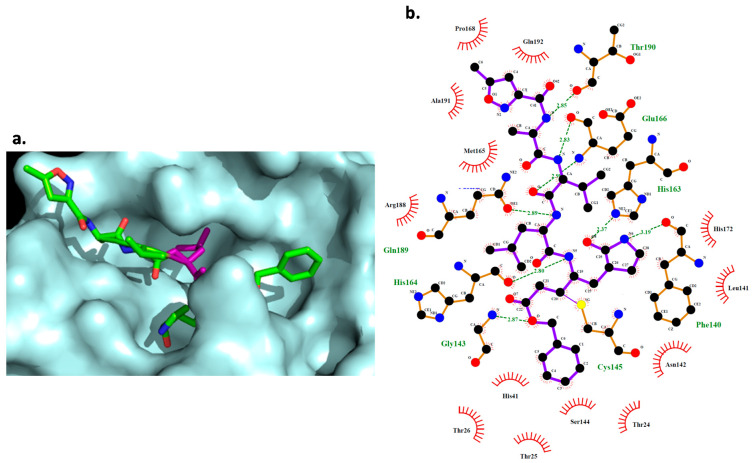
(**a**) N3 (green) interaction with the active site of Mpro (cyan). Leu residue is presented in pink. (**b**) 2D representation of the interaction between N3 and Mpro.

From the Jeon et al. [28] database, the result shows that remdesivir is the drug that interacts with more residues (18). Digoxin, bazedoxifene, and dronedarone interact with 15 residues, while thioridazine and chloroquine interact with 12. Hexachlorophene is the drug interacting with fewer residues (five). From those interactions, remdesivir forms four hydrogen bonds (HBs), digoxin and dronedarone form two HBs, and hexachlorophene and dronedarone form one HB. From the molecules taken from the screening, DB07287 and DB02213 are the ones interacting with more residues (11); DB02438 interacts with 10, DB03259 interacts with 9, while DB01980 and DB08476 interact with 8 residues. DB02438 is the one presenting the most HBs with eight, followed by DB03259 and DB02213 with three and DB01980 and DB08476 with one. DB07287 does not form any HBs. In addition, the molecular docking analysis of the known Mpro inhibitor (N3) was performed and the result shows that it interacts with 21 residues, 8 of them being HBs (Figure 6).

The active site of Mpro is a long pocket with a narrow chamber in the middle of it. During the interaction with inhibitor N3, the backbone of its peptide-like structure fits along with the active site where the side chain of a Leu residue fits in the side chamber, making the interaction stronger (Figure 6). Comparing with the studied drugs, digoxin fits in the entire pocket, but it is not able to enter the chamber due to the lack of side chains. Hexachlorophene’s most stable pose fits in the middle of the pocket, but it does not enter the side chamber either. Bazedoxifene fits in the pocket, but due to the size of the molecule, it only interacts with one end of the pocket and it is not able to reach the chamber. Dronedarone interacts with half of the pocket but it is unable to enter the chamber, the same as chloroquine. Thioridazine fits in half of the active pocket, entering the side chamber, although it is not able to make any HBs, which may explain its low affinity. Remdesivir can occupy almost all the active site, including the chamber. From the DrugBank screening, DB01980 and DB02438 fit in the pocket but are not able to enter the side chamber, while DB03259, DB02213, DB08476, and DB07287 do manage to fit in the side chamber. Although some of these drugs can enter and have a good fit in the active site of Mpro, the lack of hydrogen bonding and the fewer interactions, compared to the N3 inhibitor, may be a drawback in achieving good binding energies that can compete with natural substrates inhibiting the enzyme.

For Mpro, Asn142 and Gly143 seem to be critical residues for binding as N3, Digoxin, DB02438, and DB02213 interact with them (Figure S3 and Figure 6b). Furthermore, interactions of DB02438 and DB02213 with Asn142, Gly143, and Glu166 match pharmacophore model 5, where HBA1 interacts with Asn142, HBA2 with Glu166, and HBA3 with Gly143. In DB02438, a distance of 6.6 Å and 3.4 Å was found for HBA1–HBA3 and HBA1–HBA2, respectively. For DB02213, HBA1–HBA3 presents a distance of 5.1 Å, while HBA1–HBA2 presents a distance of 2.9 Å.

Next, PLpro was studied and the interactions were found for each drug with the active site presented in Table 5.

**Figure 7:**
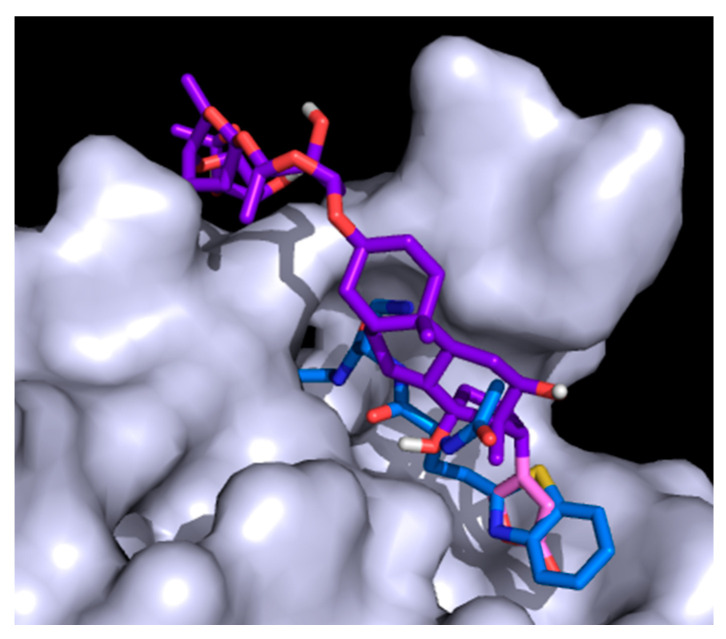
Digoxin (purple) and VIR250 (blue) interaction with PLpro (grey) active site.

Plpro has a long pocket, similar to Mpro, although it is more superficial and does not have any chambers. The main characteristic of the Plpro active site is that it closes, forming a tunnel-like structure at one end, allowing only long, thin structures to pass through it. Analyzing the poses resulting from the docking calculation, none of the drugs can pass through this tunnel. In the case of digoxin, the longest structure, it goes above this tunnel (Figure 7). The other molecules interact and fit only with the bigger end of the active site of PLpro. Looking at the interactions, dronedarone interacts with more residues (14), followed by digoxin (12), thioridazine (11), remdesivir (11), bazedoxifene (10), hexachlorophene (9), chloroquine (9), DB01980 (9), DB02213 (9), DB07287 (9), DB03259 (8), DB02438 (8), and DB08476 (8). From those, DB02438 forms five HBs; digoxin and DB02213 form four HBs; hexachlorophene and remdesivir form three HBs; bazedoxifene and DB03259 form two HBs; and dronedarone, DB01980, DB08476, and DB07287 form one HB. Thioridazine and chloroquine do not form any HBs. Comparing these results with the interaction between PLpro and experimentally found VIR250, this inhibitor presents 18 interactions, from which 6 are HBs. It seems that being able to go through the tunnel-like structure of the active site is key to have a binding affinity good enough to compete with natural peptides.

Finally, the interaction of different drugs with RdRp was evaluated, and the results are shown in Table 6.

**Figure 8:**
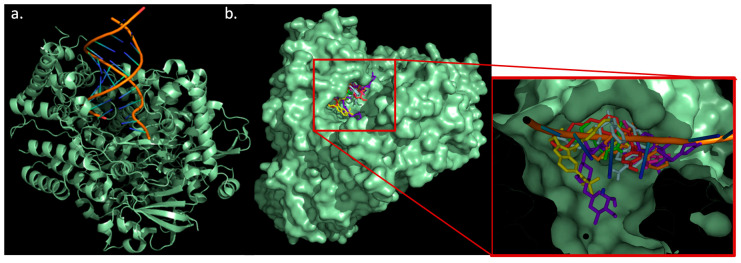
(**a**) RdRp structure. (**b**) Interaction of different drugs with RdRp.

The main function of RdRp is to catalyze the replication of RNA, using nucleotides and an RNA template. Therefore, nucleotide analogues are considered good RdRp inhibitors. The active site of RdRp is very accessible, allowing for RNA synthesis (Figure 8a). Analyzing the thirteen drugs, all of them fit perfectly in the active site (Figure 8b). Digoxin was found to form 19 interactions with the active site, followed by dronedarone (15); bazedoxifene (14); remdesivir (13); thioridazine (13); chloroquine, DB02213, and DB07287 (11); DB08476 (9); DB03259 and DB02438 (8); DB01980 (7); and hexachlorophene (6). Seven HBs were found in digoxin interaction; four HBs in remdesivir, DB02438, and DB02213; two HBs in dronedarone and bazedoxifene; and one HB in hexachlorophene, DB03259, and DB01980. The results of digoxin agree with experimental data, as it is the one with the most interactions and HBs being able to bind tightly to the active site, competing with the natural substrate. Furthermore, digoxin matches pharmacophore model 5, where HBA1 interacts with Arg555, HB2 with Ser682, and HBA3 with Thr687 (Figure S4, SM3). In this sense, there is an HBA1–HBA2 distance of 2.8 Å, HBA1–HBA3 of 7.9 Å, and HBA2–HBA3 of 8.0 Å. These results suggest that the inhibition of SARS-CoV-2 can precede by one of these three targets, with RdRp being the most likely action mechanism for the most active compound of the series (digoxin).

### 2.6. Molecular Dynamics Simulation

The results obtained from the docking analysis were further studied using molecular dynamics techniques. The quality of the simulations was assessed, observing no weird behavior on the system by graphing the box size, density, potential energy, pressure, temperature, and volume of the system against the simulation time. Furthermore, the target-ligand complex shows a stable behavior with RMSD values lower than 0.4 nm throughout the simulation in all the models.

For Mpro, digoxin, chloroquine, dronedarone, and remdesivir were compared. The RMSD for the complex and the ligand is shown in Figure S5a. For the complex, the RMSD in all cases remained stable, with a value around 0.2 nm, while for the ligand, remdesivir presents more fluctuations throughout the simulation, followed by dronedarone. Coulomb interaction energies are more negative for digoxin, while Lennard-Jones present the lowest values in dronedarone. This means that there might be a tighter interaction between digoxin and dronedarone with the active site (Figure S5b, SM3). This can be further corroborated by analyzing the HBs. For digoxin, on average, there are five HBs between the drug and the enzyme. In some parts of the simulation, HBs increase up to seven, while the lowest HB found was one. On the other hand, chloroquine is the model forming the fewest HBs with an average of one HB, two HBs being the highest and zero being the lowest (Figure S5c). Finally, the lifetimes of the main HBs extracted from their occupancy during the simulation were estimated and plotted in Figure S6 (SM3).

The results show that there is a relation between the occupancy and the lifetime. Lifetime values range from 0.37 ps to 0.91 ps, and occupancies between 2% and 48%. Dronedarone interaction with Thr25 produces the highest HB lifetime, also having the highest occupancy (48%).

The structure resulting from the docking calculation was compared with the final structure of the MD simulation. For digoxin, the structure after the simulation fits better in the pocket, with a methyl group pointing into the side chamber (Figure S7a). Chloroquine, on the other side, due to the lack of HB and strong interaction, moves out of the pocket, with only the quinolone ring having some interaction with the active site (Figure S7b).

The results of the MD simulation of PLpro showed that digoxin and chloroquine leave the active site at the end of the simulation. This may be attributed to the fact that none of the molecules entered the tunnel-like structure, so a strong interaction could not be achieved for these molecules. The behavior of bazedoxifene was studied in more detail because it is the molecule with the highest pIC_50_ that after the simulation time stays in the active site of the enzyme. Figure S8 shows the RMSD, Coulomb, Lennard-Jones energy, and HBs.

**Figure 9:**
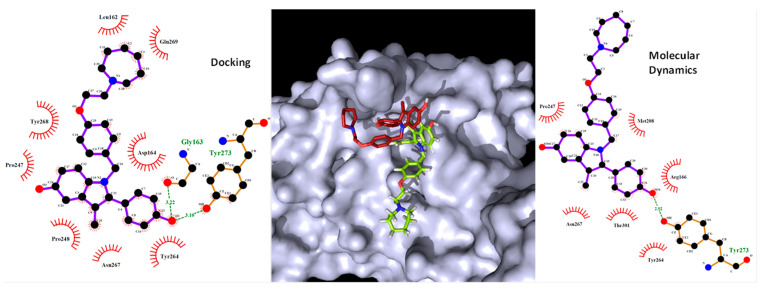
Docking (red) and MD (green) result from the interaction of bazedoxifene with PLpro.

The results show that Coulomb energy is just below zero, which indicates that although the molecule stayed in the active site, the interaction is not very strong. Again, this agrees with the HBs, where in almost all the simulations there is only one. Tyr273 is the residue making the strongest HB, with a lifetime of 0.60 ps and an occupancy of 8%. The increase in the RMSD of the ligand in the last 4 ns of simulation means more fluctuation in the drug, which may be caused by the loss of interactions with the active site. The results of the starting and the final point of the MD simulation were analyzed to see how the ligand changed throughout the simulation (Figure 9). The first important change is the loss of the HB between the drug and Gly163. Still, the HB with Tyr273 is kept along with the simulation, which is the main reason for the molecule not to leave the active site. The side chain of bazedoxifene is very flexible, and because it cannot form strong bonds with the active site, it lost interaction with Lau162 and Gln269 going outside the active site.

**Figure 10:**
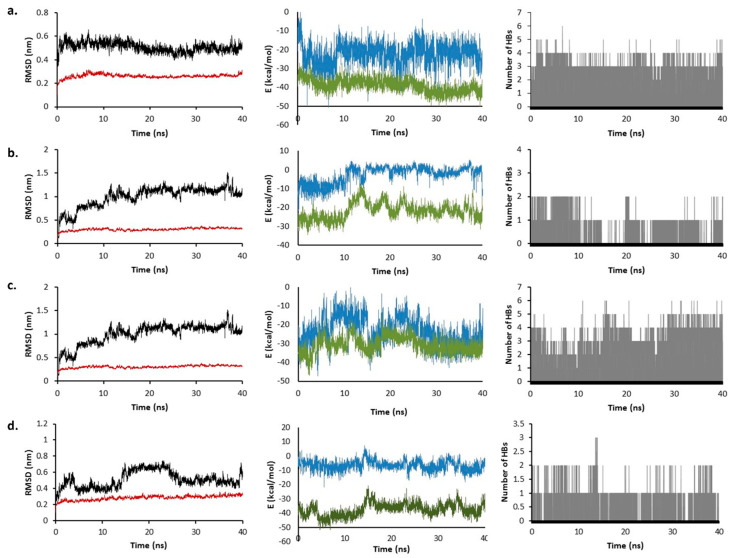
MD results for digoxin (**a**), chloroquine (**b**), remdesivir (**c**), and dronedarone (**d**) with RdRp. RMSD of the ligand is shown in black and of the protein-ligand complex in red. Coulomb interaction energy is presented in blue and Lennard-Jones in green.

For the MD simulation against RdRp, again digoxin, chloroquine, dronedarone, and remedesivir were studied. Special attention is put on remdesivir as it is a known ligand for this polymerase. The results for the interaction of the three drugs with RdRp showed that all of them stayed in the active site. Analyzing remdesivir in detail, the RMSD plot shows an average value of 0.5 nm, rising from 0.2 to 0.7 nm in the first 10 ns and stabilizing at around 0.3 nm after that. This result was compared with digoxin and dronedarone, whose RMSD values are constant throughout the simulation with a RMSD around 0.5 nm (Figure 10). This agrees with the HBs (between three and four) formed in almost the whole simulation.

**Figure 11:**
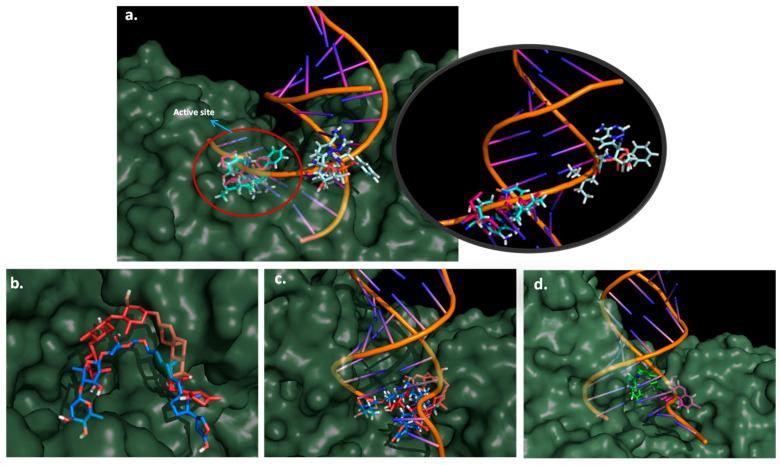
(**a**) Docking result (pink) and MD result (cyan) for the pose of remdesivir in RdRp-RNA complex. (**b**) Docking result (red) and MD result (blue) for the interaction of digoxin with RdRp active site. (**c**) RdRp-RNA complex overlapped to docking and MD results for digoxin. (**d**) RdRp-RNA complex overlapped to docking (pink) and MD (green) results for chloroquine.

**Figure 12:**
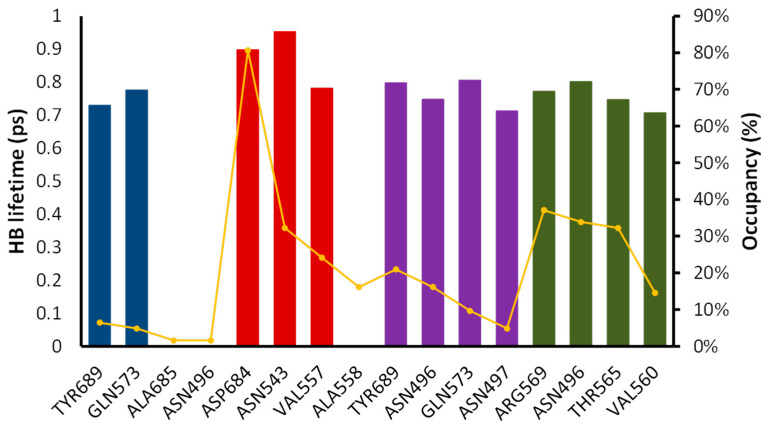
Occupancy (yellow lines) and hydrogen bond (HB) lifetimes (bars) for the MD results of RdRp. Chloroquine in blue, digoxin in red, dronedarone in purple, and remdesivir in green.

For chloroquine, RMSD values almost double compared to digoxin and remdesivir, with values around 1 nm. Coulomb and Lennard-Jones energies for digoxin are lower in all the simulations compared to chloroquine and remdesivir. This suggests that digoxin forms a tighter interaction with RdRp. Dronedarone presents a low Lennard-Jones interaction energy (comparable to digoxin), although its Coulomb energy is higher (~5 kcal/mol). As the simulation proceeds, the energy in remdesivir presents a greater fluctuation, which agrees with the RMSD plot. Analyzing remdesivir more in detail, it can be seen that in the first 10 ns, the molecule moves around 0.2 nm away from the active site. As remdesivir is moving out, HBs are less likely to be formed, and that is why between 2 and 10 ns the average HBs is two. Interestingly, although the remdesivir molecule is outside the active site, it matches with the entrance of the RNA strand (Figure 11a pale cyan structure). After this time, remdesivir is stabilized in the active site (Figure 11a, cyan structure). This might suggest that remdesivir, as a nucleotide analogue, interacts with other sites of the molecule waiting like any other nucleotide to enter the active and be incorporated to the RNA during the replication process. On the other hand, digoxin tight interactions make the molecule pose similar at the beginning and the end of the MD simulation (Figure 11b). Chloroquine interaction is weaker, as determined by the one HB that is formed in most of the simulation. Therefore, it can be seen that chloroquine moves about 10 Å inside the active site during the simulation (Figure 11d). The mechanism of digoxin may be different from remdesivir. While remdesivir substitutes a nucleotide in the RNA sequence, making further RNA transcription fail, digoxin tightly binds itself to the active site, preventing the RNA strand being replicated. Figure 11c shows how digoxin is in the same place as an RNA strand would be when interacting with RdRp. For chloroquine, there is an interaction between this drug and the active site, although the movement throughout the simulation may indicate that this is not strong enough to inhibit RNA replication. Occupancies and HB lifetimes support the aforementioned, as digoxin presents the highest lifetimes (0.95 ps) and the highest occupancy (81%), while chloroquine presents the lowest occupancy (2%) (Figure 12).

**Figure 13:**
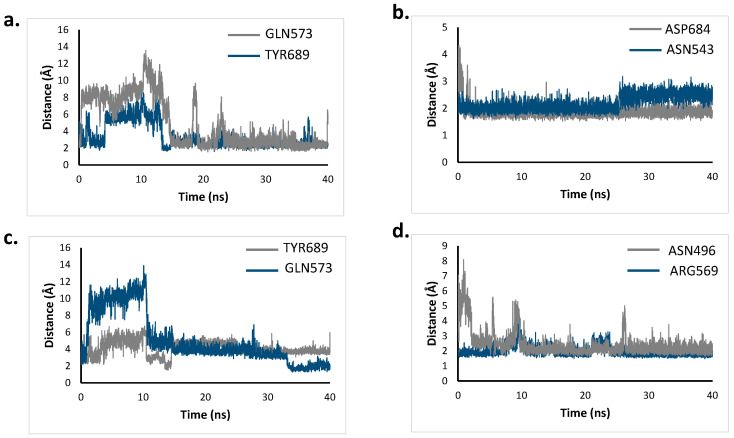
Distance between chloroquine (**a**) digoxin (**b**) dronedarone (**c**) and remdesivir (**d**) with key residues of RdRp.

Based on the highest occupancy and HB lifetimes, two key residues were chosen for the interaction of RdRp with each of the four ligands. Then, the distances between the ligand and those key residues were measured during the simulation time and plotted in Figure 13.

For the four ligands, digoxin (Figure 13b) is the one forming tighter interactions with both key residues, agreeing with its high pIC_50_ value. The average length found for digoxin with ASP684 was 1.88 Å, while for ASN543 it was 2.20 Å. The distance between ASP684 and the ligand takes around 2.5 ns to become stabilized, where a value of 4.34 Å is the highest one found. After stabilization, 2.97 Å is the longest distance between the ligand and this residue. Throughout the simulation, the distance between digoxin and ASN543 does not go above 3.18 Å, meaning that the HBs formed are very tight and stable. Analyzing RdRp’s natural substrate remdesivir, strong interactions are also formed in both residues as expected. The average distance found was 2.44 Å for ASN496 and 1.97 Å for ARG569. The HB formed between remdesivir and ARG569 is stable throughout all the simulation, while ASN496 (Figure 13d) stabilized after 10 ns. For chloroquine and dronedarone, both ligands formed interactions with GLN573 and TYR689. The average distance estimated for GLN573 was 4.85 Å for chloroquine and 5.08 Å for dronedarone. A shorter average distance was found for the interaction with TYR689 (3.35 Å for chloroquine and 4.10 Å for dronedarone). These interactions are not so strong, as seen by the greater fluctuation observed in the graph vs. the digoxin and remdesivir ones. This fact supports the lower pIC_50_ values found for both molecules. Although the distance is stabilized after 20 ns of simulation, values above the HB cutoff distance of 3.6 Å can be found in this portion of the simulation.

### 2.7. Total Binding Energy Calculations

A final step in a protein-ligand complex interaction study is to calculate the free binding energy and its van der Waals, electrostatic, and solvent-accessible surface area (SASA) contributions. MMPBSA calculations were performed on the same molecules chosen for the MD simulations. The binding energy for the PLpro, complexed with chloroquine, dronedarone, and digoxin, was not computed because those drugs did not stay in the active site of the enzyme during the MD simulation, as discussed above. For all the other complexes, binding energy and their contributions are given in Table 7.

Table 7 shows that all binding energies are negative, which suggests a favourable interaction between the different ligands and the targets. Total binding energies go from −2.77 kcal/mol to −17.55 kcal/mol in Mpro, and from −15.79 kcal/mol to −23.79 kcal/mol in RdRp. For bazedoxifene, a total binding energy value of −10.96 kcal/mol was found for PLpro, suggesting that the mechanism of this drug may be mediated by the inhibition of this protease. Dronedarone differences in its binding energy suggest a more likely polymerase mechanism than a protease one. A total energy difference of 0.15 kcal/mol was found for the interaction of chloroquine with Mpro and RdRp, which may indicate that this drug can act as a protease or polymerase inhibitor. For digoxin, it presents a 7.15 kcal/mol better total binding energy for RdRp than for Mpro. Still, its binding energy to Mpro is the second-highest, which may suggest an RdRp inhibition, but also a possible Mpro mechanism. Comparing the binding energy of Mpro known substrate N3, it is observed that chloroquine and digoxin have a stronger affinity. For RdRp, all the molecules present better binding energies than the known substrate remdesivir. Binding energies were compared to experimental pIC_50_ values, finding a very good correlation in the RdRp model with an R^2^ of 0.843. Furthermore, experimental and predicted pIC_50_ values for the four compounds studied for Mpro and RdRp were compared, and an R^2^ higher than 0.99 was found.

### 2.8. Frontier Orbital Analysis

In the last twenty years, the frontier orbitals began to play a pivotal role in the medicinal chemistry field; the studies of highest occupied molecular orbital (HOMO) and lowest unoccupied molecular orbital (LUMO) have allowed the understanding of several biological activities, such as antimicrobial [62,63,64,65], anticancer [66,67,68,69,70,71,72,73], antifungal [74,75,76], and cytotoxicity [77,78,79,80]. More recently, the shape of frontier orbitals has been used to differentiate between active and inactive compounds against a specific disease [81,82].

**Figure 14:**
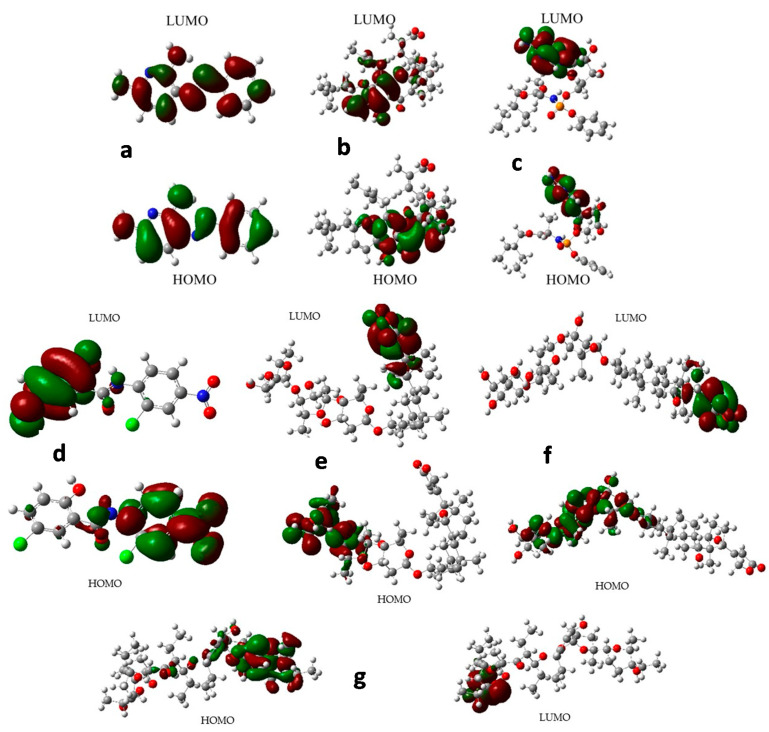
Frontier orbital for (**a**) phenazopyridine; (**b**) dihydrogambomic acid; (**c**) remdisivir; (**d**) Niclosamide; (**e**) Digitoxin; (**f**) digoxin; (**g**) salinomycin. HOMO: highest occupied molecular orbital. LUMO: lowest unoccupied molecular orbital.

In this line, the shape and energy of HOMO and LUMO orbitals have been calculated with the aim of finding any hint that allows us to discriminate between the more active and the least active compounds against COVID-19. In this sense, more and less active compounds from the data in Table S3 were selected to carry out the frontier orbital analysis. According to Figure 14, compounds with the least activity (Figure 14a) have both HOMO and LUMO scattered under almost the entire molecular regions, whereas dihydrogambomic acid (Figure 14b) and remdisivir (Figure 14c) present their frontier orbital located in the same molecular region. On the other hand, the most active compounds have the frontier orbitals located in a specific, but different, molecular region (Figure 14d–g). Therefore, these results suggest that the acceptor-donor characteristic of these compounds plays a fundamental role in the inhibition of SARS-CoV-2. The results contrast with some reported before, where the antiviral compound showed frontier orbital located in the same region of the molecule, as explained above for remdesivir [83,84]. It is important to remark that for compounds with pIC_50_ values in the 5–6 interval, a clear tendency concerning the molecular orbital shapes is not observed; furthermore, they are not shown herein.

**Figure 15:**
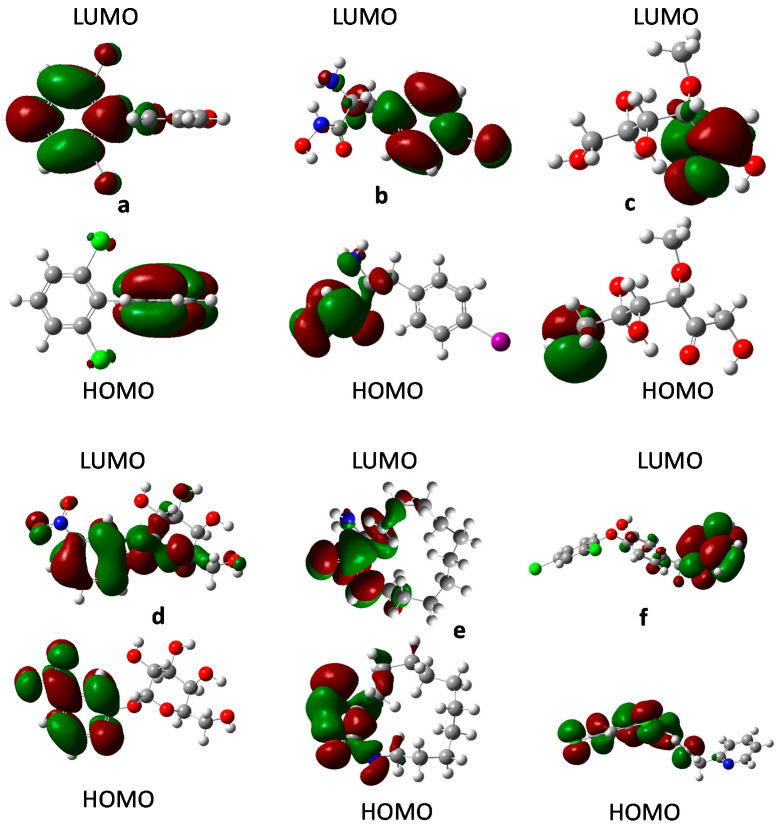
Frontier orbital for (**a**) DB03259; (**b**) DB01980; (**c**) DB02438; (**d**) DB02213; (**e**) DB08476; (**f**) DB07287.

In order to verify if the aforementioned pattern is met for the most prominent compounds with a potential anti-COVID-19 activity from the screening, the molecular orbitals for the six compounds chosen for the docking were computed, and the results are presented in Figure 15. According to this, all compounds met this characteristic except for DB08476 and DB02213. For DB08476, the HOMO and LUMO are in the same region of the molecule. In this case, that makes sense because all the functional groups that will be responsible for the main interactions are located in the same region. For DB02213, while the HOMO occupied one molecular region as seen for other active molecules, the LUMO is present in the central region of the molecule sharing some regions with the HOMO. Still, our results suggest that the structural requirement for COVID 19-active compounds may lie not only in the molecular descriptors described in M13, but also in the frontier molecular shape.

## 3. Materials and Methods

### 3.1. Datasets Preparation

The dataset used in this study is composed of 44 compounds extracted from the FDA-approved drugs in Joan et al.’s study, which were experimentally tested as possible antiviral candidates in the treatment of COVID-19 [28]. This dataset is diverse in structure and adequate for the construction of a QSAR model. The 2D structures of all compounds were downloaded from PubChem (https://pubchem.ncbi.nlm.nih.gov/ (accessed on 18 February 2021)). Three-dimensional structures were optimized at the Universal Force Field (UFF) level of theory using the RDKit (https://www.rdkit.org/ (accessed on 18 February 2021)), implemented in the QuBiLS-MAS software [85]. The dependent variable used in this study is the experimental value of the half-maximal inhibitory concentration [pIC_50_ = −log (IC_50_, M)]. A rational division of the dataset into training (80%) and test sets (20%) was performed by employing a K-means clustering analysis, which has been demonstrated to be more acceptable for an appropriate assessment of the predictability of the models [86]. The clustering analysis was performed on the standardized variables, the Euclidean distance as a similarity metric, and 500 iterations. Then, around 20% of the molecules of each cluster were selected as a test set.

In addition, the drugs included in the recent version of the DrugBank database [29] (Version 5.1.7) were downloaded from the webpage (https://www.drugbank.ca/ (accessed on 18 February 2021)) and used in the present study for a virtual screening prospective analysis by taking into account the datasets labelled as Approved (2636 compounds), Experimental (6127 compounds), Nutraceutical (118 compounds), and Withdrawn (245 compounds). All these datasets were cleaned by the removal of inorganic, organometallic, and mixture compounds, while salts were neutralized. The AD was evaluated in these compounds and the ones classified as IN were considered for the prediction of the pIC_50_ value.

### 3.2. Descriptors Calculations and Modelling Process

Two types of descriptors were calculated, topographical 2D and 3D. For the calculation of the 2D descriptors, QuBiLS-MAS software [85] was employed, while for the estimation of the 3D descriptors, QuBiLS-MIDAS software [87] was used. Then, 945 2D and 114 3D descriptors were calculated and used for the modelling process. These types of topographical descriptors have demonstrated to be adequate for QSAR model construction in different applications, such as toxicology [88], hepatotoxicity [89], antimalarial [90], and others [91,92,93].

Modelling considering only 2D descriptors, only 3D descriptors, and the combination 2D-3D descriptors was built. In a first stage, a supervised attribute selection using the Genetic Algorithms was applied to determine small subsets of attributes, which contain the major information content about the pIC_50_, taking into account the ratio molecules: descriptors 5:1 [94]. This analysis was performed using MATLAB version R2014b (Mathworks Inc. software, Natick, MA, USA) [95]. The selected subsets were used for the model construction, employing the multiple linear regression approach.

### 3.3. Domain of Applicability

The analysis of the AD (applicability domain) of the model is an important way to measure how reliable the prediction of the pIC_50_ value is. The applicability domain of a model consists of the evaluation of the theoretical spatial region, as defined by the descriptors involved in a model. Although there is not a method universally considered the best for determining the AD [96], there are some recognized methods for this analysis, such as the one based on the principal component analysis (PCA) range, the Euclidean distance, citi-block distance, and probability density, which are implemented in AMBIT discovery and used in the present study [35]. The PCA method is based on the analysis of the range of each descriptor, and if at least one of the descriptors of a new molecule is not in the range, then it is considered as OUT of the AD. In the Euclidean and City block methods, distances are determined as a cut-off value of the corresponding distance from the centroid to determine if the compound is OUT or IN. In the probability density method, a standard distribution is constructed using parametric methods, being able to identify interior empty regions in the dataset distribution. The consensus approach was applied to take a decision by taking into account that if a molecule is catalogued as OUT by two or more AD methods, then it can be finally considered OUT [89].

### 3.4. Model Performance

The model performance was evaluated by the traditional statistical analysis parameters:

- The collinearity between the descriptors was evaluated by taking into account a Pearson’s correlation coefficient of <0.7.
- The coefficient of determination (R^2^) is used as a global evaluator of the model.
- The predictability of the model is determined by the leave one out cross-validation coefficient (Q^2^_LOO_), the external validation coefficient (Q^2^_ext_), and the bootstrapping coefficient (Q^2^_boot_).
- The robustness of the model is evaluated in terms of the Y-scrambling analysis (a(R^2^) and a(Q^2^)), based on a random perturbation of the Y values.
- Additionally, the linear fitting of the model was evaluated in terms of the Fisher coefficient value (F) and the residual standard deviation (s).
- Tropsha’s test analysis is performed for the leave-one-out and external validation analysis, as suggested by the Organization for Economic Cooperation and Development (OECD) principles [42].

### 3.5. Pharmacophore Modelling

A ligand-based pharmacophore modelling was performed using the four most active compounds extracted from the Jeon et al. [28] database and PharmaGist web server [97,98]. The molecular and electronic features derived from the calculation were used to build pharmacophore models. Then, each model was screened against the previously cleaned approved, experimental, nutraceutical, and withdrawn DrugBank databases using Pharmit [99]. Finally, the molecules that fit the pharmacophore were compared to their predicted pIC_50_ values.

### 3.6. Molecular Docking

For the docking studies, 3 enzymes were chosen as biological targets based on their importance in COVID-19, as described in a recent publication [3]. The targets chosen were SARS-CoV-2 Mpro, PLpro, and RdRp, which are key for the virus life cycle. Ligands were obtained from the DrugBank database [29], based on the Jeon et al. dataset [28], and the DrugBank screening, while the enzymes were taken from the Protein Data Bank [100]. For Mpro, the X-ray crystal structure of the enzyme in complex with an inhibitor N3 [101] was chosen (PDB: 6LU7). For PLpro, the crystal structure of the enzyme in complex with a peptide inhibitor VIR250 (PDB: 6WUU) was selected. Finally, for RdRp, the electron microscopy structure of the nsp12-nsp7-nsp8 complex bound to the template primer RNA and the triphosphate form of Remdesivir (PDB: 7BV2) was used. To prepare the structure, Pymol [102] was employed to eliminate water molecules, dimers, natural ligands, and other molecules such as ions. Then, Autodock Tools [103] was used to eliminate non-polar hydrogen atoms, to identify rotating bonds, and to transform to the correct format (.PDBQT). Calculations were performed using autodock-VINA [104]. For the calculation parameters, spacing was set to 1 A; default exhaustiveness and full flexibility of the ligands were chosen. The grid box for each enzyme is presented in the supplementary information (SM3, Table S7). Docking assessment was performed as a validation procedure using the docking accuracy strategy. The results were analyzed using Pymol [102], Ligplus+ [105], and Discovery studio visualizer [106].

### 3.7. Molecular Dynamic Simulations

Further classical molecular dynamic (MD) simulations were performed based on experimental pIC_50_. This type of calculation allows us to study the relative stability of the ligand of interest in the active site of the enzyme, gaining insights on different parameters such as hydrogen bond formation. For the calculation, the already cleaned enzyme plus the ligand obtained from the docking calculations were used. The system was solvated using the three-point water model (TIP3), in a cube shape. Sodium or chlorine atoms were used as appropriate to neutralize the charge of the system. Enzyme topology was built using Gromacs 2019 [107], and ligand topology with CHARMM General Force Field (CGenFF) [108]. Calculations were performed with Gromacs 2019 [107] using the Chemistry at Harvard Macromolecular Mechanics (CHARMM) forcefield. First, the system was relaxed and then equilibrated using a constant number of particles, pressure, and temperature (NVT) for 100 ps at 300 K, using Berendsen thermostat temperature coupling [109] before starting the dynamic. For the simulation parameters, thermostat coupling was set to 300 K, pressure coupling at 1 Barr and the simulation ran for 40 ns (40,000 steps of 1ps each) [58].

### 3.8. Free Energy Calculations

Binding free energy calculations were performed using the Molecular Mechanics Poisson–Boltzmann Surface Area (MM-PBSA) approach with the *g_mmpbsa* tool [110]. Binding free energy is obtained according to Equation (1), taking into account the vacuum Molecular Mechanics (MM) potential energy for non-bonded and bonded interactions (E_MM_), plus the polar (G_polar_) and non-polar (G_nonpolar_) solvation energy (Equation (2)).

G_X_ = E_MM_ + G_polar_ + G_nonpolar_ (2)

X = protein, ligand, complex.

MM forcefield parameters are used to calculate E_MM_. G_polar_ is obtained by solving the Poisson–Boltzmann equation, while G_nonpolar_ is based on the Solvent Accessible Surface Area (SASA) model. The three parameters were extracted between 5 and 40 ns of the MD simulation trajectory by taking snapshots every 2 ns.

### 3.9. Frontier Orbital Analysis

The main purpose of this section was to investigate whether or not the frontier orbitals shapes could allow qualitatively distinguishing structures with high vs. low anti-COVID-19 activity. For this purpose, the selected structures were optimized at the DFT theory level with the WB97XD/6-311G(d,p) [82,93,111,112] method, implemented in Gaussian 16 [113] for Linux. Following optimization, the .chks files were retrieved and used to generate the frontier molecular orbitals with a 0.02 au isosurface.

## 4. Conclusions

For the QSAR analysis, 2D structures of the compounds were used as downloaded from the PubChem website, and 3D structures were satisfactorily optimized at the UFF level of theory. A robust model for pIC_50_ prediction with only five attributes was obtained with good statistical parameters (R^2^ = 0.897, Q^2^_LOO_ = 0.854 and Q^2^_ext_ = 0.876, among others). The model has been demonstrated to be robust enough for the prediction of biological activity with a wide applicability domain, making it possible to predict pIC_50_ values for more than 4000 compounds extracted from the Drugbank database. Based on their pIC_50_ values, it was possible to identify 57 possible potent drugs, which can be used for the treatment of COVID-19. From these, six compounds, i.e., Para-iodo-d-phenylalanine hydroxamic acid (DB01980), 2′,6′-Dichloro-Biphenyl-2,6-Diol (DB03259), 3-*O*-Methylfructose (DB02438), Metanitrophenyl-Alpha-d-Galactoside (DB02213), 3-amino-azacyclotridecan-2-one (DB08476), and 2-(2,4-dichlorophenoxy)-5-(pyridin-2-ylmethyl)phenol (DB07287), were found as potential candidates due to their activity (pIC_50_ > 6.6) and are suggested for further experimental studies. Interestingly, several antifungal and antibiotic compounds such as miconazole, amikacin, streptomycin, lincomycin, and spiramycin were found to be potent SARS-CoV-2 inhibitors, the same as monensin, flunisolide, fluticasone propionate, and tixocortol, which are used to treat bronchitis, asthma, and allergic rhinitis. From the docking analysis, the affinity scores found for the interaction of the studied drugs with the three enzymes were all negative, with values from −4.5 to −9.4 kcal/mol, suggesting, at first sight, that all the molecules could inhibit the biological targets. Further analysis showed that the Mpro binding pocket has a side chamber where a Leu residue of inhibitor N3 enters. Several of the studied compounds manage to fit in this chamber, although none of them can obtain the same number of interactions and HBs as N3. On PLpro, a tunnel-like structure on the active site where none of the studied molecules entered made drug interaction with the active site not very strong. The best results were obtained for RdRp, where digoxin has 19 interactions, including 7 HBs. The molecular dynamic simulation for the selected compounds contributed substantially to the understanding of the protein-ligand interaction for the three considered targets. MD simulation showed that in Mpro, digoxin accommodates better in the pocket with a methyl group pointing to the chamber. For PLpro, digoxin and chloroquine failed to stay in the active site after the simulation. Again, RdRp-drug complexes were found to be the better option after the MD simulation. Good fit with the active site and an overlay when adding the experimental RNA structure suggest RdRp as a plausible mechanism of several studied drugs. MMPBSA calculation showed that all values are negative, meaning that there is a favourable interaction between the studied drugs and the chosen targets. For RdRp, digoxin was found to have the highest affinity, matching experimental pIC_50_ values. The frontier molecular orbitals analysis suggests that it is possible to separate the compounds based on the molecular orbital shapes.

## Figures and Tables

**Table 1:** Statistical parameters for model M13 obtained by the 2D-3D modeling and Tropsha’s test results.

N° of Attributes	R^2^	Q^2^_LOO_		Q^2^_LMO_		Q^2^_boot_		Q^2^_ext_
5	0.897	0.854		0.834		0.829		0.876
a(R^2^)	a(Q^2^)	Kx		Kxy		F		s
0.104	−0.328	0.259		0.287		50.61		0.160
**Tropsha’s Test**								
	**Leave-One-Out Validation**				**External Validation**			
**Criterion**	**Result**		**Assessment**		**Result**		**Assessment**	
R^2^ > 0.6	0.897		PASS		0.897		PASS	
R^2^_Val_ * > 0.5	0.854		PASS		0.876		PASS	
(R^2^_Val_ − R_0_^2^)/R^2^_Val_ < 0.1	0.001		PASS		0.002		PASS	
(R^2^_Val_ − R_0_′^2^)/R2Val < 0.1	0.021		PASS		0.020		PASS	
abs(R_0_^2^ − R_0_′^2^) < 0.1	0.018		PASS		0.016		PASS	
0.85 < k < 1.15	1.001		PASS		1.017		PASS	
0.85 < k′ < 1.15	0.998		PASS		0.982		PASS	

* The label “Val” refers to the leave-one-out (LOO) or external (EXT) validation.

**Table 2:** Datasets extracted from the DrugBank 5.1.7 (https://www.drugbank.ca/ (accessed on 18 February 2021)) and used for the prospective screening.

Database	N° Total of Molecules	N° Molecules IN	Coverage (%)	pIC_50_ Range (Only Molecules IN)
Approved	2265	1882	83	3.68–7.10
Experimental	5858	4519	77	3.58–7.51
Nutraceutical	102	55	54	3.99–5.85
Withdrawn	228	208	91	4.24–7.15

**Table 3:** DrugBank screening using different pharmacophore models.

Model	Features	Number of Hits
Model 1	HBA1, HBA2, HBA3, HPH4	1
Model 2	HBA2, HBA3, HPH4	11
Model 3	HBA1, HBA3, HPH4	16
Model 4	HBA1, HBA2, HPH4	48
Model 5	HBA1, HBA2, HBA3	45

**Table 4:** Interactions of different drugs with Mpro.

Drug	Interactions
Digoxin	Thr24, Thr25, Thr26, Thr45, Met49, Leu141, **Asn142, Gly143**, Cys145, His164, Met165, Glu166, Pro168, Gln189, Thr190
Hexachlorophene	Met49, Cys145, **His164**, Met165, Glu166
Bazedoxifene	Thr25, Thr26, Leu27, His41, Met49, **Tyr54**, Asn142, Gly143, Cys145, His164, Met165, Gln166, **Asp187**, Arg188, Gln189,
Dronedarone	Thr25, **Thr26**, Met49, Phe140, Leu141, Asn142, Gly143, Cys145, His163, Met165, Glu166, Pro168, Gln189, Thr190, Ala191
Thioridazine	His41, Asn142, Met49, Tyr54, Leu141, His164, Met165, Glu166, Asp187, Arg188, Gln189, Thr190
Chloroquine	His41, Met49, Tyr54, Cys145, His164, Met165, Glu166, Asp187, Arg188, Gln189, Thr190, Gln192
Remdesivir	His41, Thr25, Thr26, Leu27, Met49, Phe140, **Leu141**, Asn142, **Gly143**, **Ser144**, Cys145, **His163**, His164, Met165, Glu166, Asp187, Arg188, Gln189
DB03259	His41, Asn142, Gly143, **Ser144**, **His163**, **Cys145**, His164, Met165, Gln189
DB01980	**His41**, Asn142, Cys145, His164, Met165, Glu166, Arg188, Gln189
DB02438	**Phe140**, **Leu141**, **Asn142**, **Gly143**, **Ser144**, **Cys145**, **His163**, His164, Met165, **Glu166**
DB02213	His41, **Leu141**, **Asn142**, **Gly143**, Cys145, His164, Met165, **Glu166**, Asp187, Arg188, Gln189
DB08476	**His41**, Cys145, His164, Met165, Glu166, Asp187, Arg188, Gln189
DB07287	His41, Met49, Tyr54, His164, Met165, Glu166, Pro168, Asp187, Gln189, Thr190, Ala191

Hydrogen bonds in bold.

**Table 5:** Interactions of different drugs with PLpro.

Drug	Interactions
Digoxin	Asp108, **Asn109**, **Gly160**, Glu161, **Leu162**, Gly163, **Asp164**, Pro248, Tyr264, Tyr268, Tyr273, Thr301
Hexachlorophene	Leu162, **Asp164**, Gly163, Pro248, Tyr264, Tyr268, Gln269, **Gly271**, **Tyr273**
Bazedoxifene	Leu162, **Gly163**, Asp164, Pro247, Pro248, Tyr264, Asn267, Tyr268, Gln269, **Tyr273**
Dronedarone	Leu162, Gly163, Asp164, **Arg166**, Met208, Ser245, Ala246, Pro248, Gly266, Tyr264, Tyr268, Tyr273, Thr301, Asp302
Thioridazine	Leu162, Gly163, Asp164, Pro247, Pro248, Tyr264, Gly266, Asn267, Tyr268, Tyr273, Thr301
Chloroquine	Leu162, Gly163, Asp164, Pro248, Tyr264, Tyr268, Gln269, Tyr273, Thr301
Remdesivir	Leu162, Gly163, **Asp164**, **Arg166**, Glu167, Met208, Pro248, Tyr264, Tyr268, Gly271, **Tyr273**
DB03259	Gly163, Asp164, **Arg166**, Pro248, Tyr264, Tyr268, **Tyr273**, Thr301
DB01980	Asp164, Arg166, Pro247, Pro248, Tyr264, Tyr268, **Tyr273**, Thr301, Asp302
DB02438	**Asp164**, **Arg166**, Pro247, Pro248, Tyr264, **Tyr273**, **Thr301**, **Asp302**
DB02213	Leu162, Gly163, Asp164, Glu167, Pro248, **Tyr264**, **Tyr268**, **Tyr273**, **Thr301**
DB08476	Asp164, Met208, Pro247, Pro248, Tyr264, Tyr268, **Tyr273**, Thr301
DB07287	Leu162, Gly163, Asp164, Arg166, Tyr264, Tyr268, **Tyr273**, Thr301, Asp302

Hydrogen bonds in bold.

**Table 6:** Interactions of different drugs with RdRp.

Drug	Interactions
Digoxin	Lys500, **Ser501**, Asn543, **Arg555**, Val557, Ala558, Gly559, Val560, Thr565, **Arg569**, **Ser682**, Gly683, Asp684, **Ala685**, **Thr687**, **Ala688**, Asn691, Ser759, Thr868
Hexachlorophene	Ser501, **Thr565**, Gly559, Val560, Arg 569, Gly683,
Bazedoxifene	**Asn496**, Asn497, Lys500, Ser501, Val560, Ser561, Ile562, **Thr565**, Arg569, Gln573, Leu576, Lys577, Gly683, Tyr689
Dronedarone	Asn496, Asn497, Ser501, Val560, Thr565, Arg569, Gln573, Leu576, Lys577, Gly683, Asp684, Ala685, Thr687 **Ala688**, **Tyr689**
Thioridazine	Lys500, Ser501, Ala512, Asn543, Val557, Ala558, Gly559, Ile562, Thr565, Arg569, Gly683, Asp684, Ala685
Chloroquine	Asn496, Asn497, Ser501, Ala502, Val560, Ser561, Ile562, Thr565, Agr569, Gly683, Ala685,
Remdesivir	**Asn497**, Val495, Asn496, Lys500, **Ser501**, Val560, **Thr565**, **Arg569**, Gln573, Leu576, Lys577, Gly683, Ala685
DB03259	Val410, **Gln444**, Ala547, Ile548, Ser549, Arg553, Ala554, Arg555
DB01980	Thr565, **Arg569**, Gln573, Leu576, Lys577, Ala685, Tyr689
DB02438	Lys500, **Ser501**, Gly559, **Val560**, Ser561, Ile562, **Thr565**, **Arg569**
DB02213	Val410, **Phe440**, Phe441, Gln444, **Lys545**, Ile548, Ser549, Lys551, **Arg553**, Ala554, **Arg555**
DB08476	Asp452, Arg553, Arg555, Thr556, Asp623, Arg624, Thr680, Ser682, Asn691
DB07287	Lys500, Ser501, Ala512, Thr565, Arg569, Gln573, Leu576, Lys577, Gly683, Ala685, Tyr689

Hydrogen bonds in bold.

**Table 7:** Energy contributions to the total binding energy of the protein-ligand interaction in the three evaluated targets.

Protein	Compound	van der Waals (kcal/mol)	Electrostatics (kcal/mol)	SASA (kcal/mol)	Total Binding Energy (kcal/mol)	pIC_50 experimental_	pIC_50_ _predicted_
MPro	Chloroquine	−26.89	−1.71	−3.10	−17.55	5.14	5.15
	Digoxin	−43.41	−15.82	−5.47	−16.64	6.72	6.68
	Dronedarone	−11.21	−1.30	−1.28	−4.92	5.41	5.46
	Remdesivir	−14.17	−3.05	−1.81	−2.77	4.94	4.87
	N3	−44.96	−8.74	−5.67	−15.99	--	--
PLpro	Bazedoxifene	−16.72	−1.43	−1.89	−10.96	5.46	5.57
RdRp	Chloroquine	−24.21	−1.49	−3.37	−17.70	5.14	5.15
	Digoxin	−46.28	−18.53	−6.06	−23.79	6.72	6.68
	Dronedarone	−41.18	−1.88	−5.82	−20.97	5.41	5.46
	Remdesivir	−35.98	−16.80	−4.85	−15.79	4.94	4.87

## Data Availability

Not applicable.

## Supplementary Materials

The following are available online, Figure S1. Pearson’s coefficient range for each descriptor concerning the other descriptors; Figure S2. a. Pharmacophore model 4 fitting DB03259. b. Pharmacophore model 5 fitting DB02438; Figure S3. 2D representation of the interaction of Digoxin (a), DB02438 (b), and DB02213 (c) with the active site of Mpro; Figure S4. 2D representation of the interaction of Digoxin with the active site of RdRp; Figure S5. MD results for Mpro. a. RMSD of the protein-ligand complex (red) and the ligand (black). b. Coulomb (blue) and Lennard-Jones (green) interaction energy. c. the number of hydrogen bonds; Figure S6. Occupancy (yellow line) and HB lifetimes (Bars) for the MD results of Mpro. Chloroquine in blue, digoxin in red, dronedarone in purple, and remdesivir in green; Figure S7. Interaction of MPro (pale cyan) with a. Docking (yellow) and MD (green) results for Digoxin. b. Docking (pink) and MD (orange) result for Chloroquine; Figure S8. MD results for PLpro interaction with bazedoxifene. a. RMSD of the protein-ligand complex (red) and the ligand (black). b. Coulomb (blue) and Lennard-Jones (green) interaction energy. c. the number of hydrogen bonds; Table S1. Models obtained for the 2D, 3D, and combined modeling; Table S2. DockCov2 scores of compounds in the Jeon et al. database; Table S3. Jeon et al. database screening using pharmacophore models 4 and 5; Table S4. Best compounds for the DrugBank screening using pharmacophore models 4 and 5; Table S5. Docking results of the Dataset against Mpro, PLpro, and RdRp, ordered from largest to lowest pIC_50;_ Table S6. Docking results of the Best Molecules of the DrugBank screening, ordered from largest to loweest predicted pIC_50_; Table S7. Gris box used for the docking study.

## Sample Availability

Samples of the compounds are not available from the authors.

## References

[1] Zhu Y., Li J., Pang Z. (2020). Recent insights for the emerging COVID-19: Drug discovery, therapeutic options and vaccine development. Asian J. Pharm. Sci..

[2] Tahir ul Qamar M., Alqahtani S.M., Alamri M.A., Chen L.L. (2020). Structural basis of SARS-CoV-2 3CLpro and anti-COVID-19 drug discovery from medicinal plants. J. Pharm. Anal..

[3] Gil C., Ginex T., Maestro I., Nozal V., Barrado-Gil L., Cuesta-Geijo M.Á., Urquiza J., Ramírez D., Alonso C., Campillo N.E. (2020). COVID-19: Drug Targets and Potential Treatments. J. Med. Chem..

[4] Hatada R., Okuwaki K., Mochizuki Y., Handa Y., Fukuzawa K., Komeiji Y., Okiyama Y., Tanaka S. (2020). Fragment Molecular Orbital Based Interaction Analyses on COVID-19 Main Protease—Inhibitor N3 Complex (PDB ID: 6LU7). J. Chem. Inf. Model..

[5] Eastman R.T., Roth J.S., Brimacombe K.R., Simeonov A., Shen M., Patnaik S., Hall M.D. (2020). Remdesivir: A Review of Its Discovery and Development Leading to Emergency Use Authorization for Treatment of COVID-19. ACS Cent. Sci..

[6] Xiu S., Dick A., Ju H., Mirzaie S., Abdi F., Cocklin S., Zhan P., Liu X. (2020). Inhibitors of SARS-CoV-2 Entry: Current and Future Opportunities. J. Med. Chem..

[7] Ahidjo B.A., Loe M.W.C., Ng Y.L., Mok C.K., Chu J.J.H. (2020). Current Perspective of Antiviral Strategies against COVID-19. ACS Infect. Dis..

[8] Korkmaz B., Lesner A., Marchand-Adam S., Moss C., Jenne D.E. (2020). Lung Protection by Cathepsin C Inhibition: A New Hope for COVID-19 and ARDS?. J. Med. Chem..

[9] Lu L., Zhong W., Bian Z., Li Z., Zhang K., Liang B., Zhong Y., Hu M., Lin L., Liu J. (2020). A comparison of mortality-related risk factors of COVID-19, SARS, and MERS: A systematic review and meta-analysis: Mortality-related risk factors of COVID-19, SARS, and MERS. J. Infect..

[10] Yuan X., Yang C., He Q., Chen J., Yu D., Li J., Zhai S., Qin Z., Du K., Chu Z. (2020). Current and Perspective Diagnostic Techniques for COVID-19. ACS Infect. Dis..

[11] Barros R.O., Junior F.L., Pereira W.S., Oliveira N.M., Ramos R.M. (2020). Interaction of Drug Candidates with Various SARS-CoV-2 Receptors: An in Silico Study to Combat COVID-19. J. Proteome Res..

[12] Gao K., Nguyen D.D., Chen J., Wang R., Wei G.W. (2020). Repositioning of 8565 Existing Drugs for COVID-19. J. Phys. Chem. Lett..

[13] Zeng X., Song X., Ma T., Pan X., Zhou Y., Hou Y., Zhang Z., Li K., Karypis G., Cheng F. (2020). Repurpose Open Data to Discover Therapeutics for COVID-19 Using Deep Learning. J. Proteome Res..

[14] Chien M., Anderson T.K., Jockusch S., Tao C., Li X., Kumar S., Russo J.J., Kirchdoerfer R.N., Ju J. (2020). Nucleotide Analogues as Inhibitors of SARS-CoV-2 Polymerase, a Key Drug Target for COVID-19. J. Proteome Res..

[15] Saul S., Einav S. (2020). Old drugs for a new virus: Repurposed approaches for combating COVID-19. ACS Infect. Dis..

[16] Weiss C., Carriere M., Fusco L., Fusco L., Capua I., Regla-Nava J.A., Pasquali M., Pasquali M., Pasquali M., Scott J.A. (2020). Toward Nanotechnology-Enabled Approaches against the COVID-19 Pandemic. ACS Nano.

[17] Ghosh A.K., Brindisi M., Shahabi D., Chapman M.E., Mesecar A.D. (2020). Drug Development and Medicinal Chemistry Efforts toward SARS-Coronavirus and Covid-19 Therapeutics. ChemMedChem.

[18] Batra R., Chan H., Kamath G., Ramprasad R., Cherukara M.J., Sankaranarayanan S.K. (2020). Screening of Therapeutic Agents for COVID-19 Using Machine Learning and Ensemble Docking Studies. J. Phys. Chem. Lett..

[19] Asai A., Konno M., Ozaki M., Otsuka C., Vecchione A., Arai T., Kitagawa T., Ofusa K., Yabumoto M., Hirotsu T. (2020). COVID-19 drug discovery using intensive approaches. Int. J. Mol. Sci..

[20] Wang J. (2020). Fast Identification of Possible Drug Treatment of Coronavirus Disease-19 (COVID-19) through Computational Drug Repurposing Study. J. Chem. Inf. Model..

[21] Kandeel M., Al-Nazawi M. (2020). Virtual screening and repurposing of FDA approved drugs against COVID-19 main protease. Life Sci..

[22] (2020). ISRCTN83971151 Public Health Emergency SOLIDARITY Trial of Treatments for COVID-19 Infection in Hospitalized Patients. http://www.who.int/trialsearch/Trial2.aspx?TrialID=ISRCTN83971151.

[23] WHO (2020). Solidarity Trial Consortium Repurposed Antiviral Drugs for Covid-19—Interim WHO Solidarity Trial Results. N. Engl. J. Med..

[24] Lou Y., Liu L., Yao H., Hu X., Su J., Xu K., Luo R., Yang X., He L., Lu X. (2021). Clinical Outcomes and Plasma Concentrations of Baloxavir Marboxil and Favipiravir in COVID-19 Patients: An Exploratory Randomized, Controlled Trial. Eur. J. Pharm. Sci..

[25] Caly L., Druce J.D., Catton M.G., Jans D.A., Wagstaff K.M. (2020). The FDA-approved drug ivermectin inhibits the replication of SARS-CoV-2 in vitro. Antivir. Res..

[26] Elnagdy S., AlKhazindar M. (2020). The Potential of Antimicrobial Peptides as an Antiviral Therapy against COVID-19. ACS Pharmacol. Transl. Sci..

[27] Yan V.C., Muller F.L. (2020). Advantages of the Parent Nucleoside GS-441524 over Remdesivir for Covid-19 Treatment. ACS Med. Chem. Lett..

[28] Jeon S., Ko M., Lee J., Choi I., Byun S.Y., Park S., Shum D., Kim S. (2020). Identification of antiviral drug candidates against SARS-CoV-2 from FDA-approved drugs. Antimicrob. Agents Chemother..

[29] Wishart D.S., Feunang Y.D., Guo A.C., Lo E.J., Marcu A., Grant J.R., Sajed T., Johnson D., Li C., Sayeeda Z. (2018). DrugBank 5.0: A major update to the DrugBank database for 2018. Nucleic Acids Res..

[30] Information N.C. For B. Digoxin. https://pubchem.ncbi.nlm.nih.gov/compound/Digoxin.

[31] Johansson S., Lindholm P., Gullbo J., Larsson R., Bohlin L., Claeson P. (2001). Cytotoxicity of digitoxin and related cardiac glycosides in human tumor cells. Anticancer. Drugs.

[32] Resham K., Patel P.N., Thummuri D., Guntuku L., Shah V., Bambal R.B., Naidu V.G.M. (2015). Preclinical drug metabolism and pharmacokinetics of salinomycin, a potential candidate for targeting human cancer stem cells. Chem. Biol. Interact..

[33] National Center for Biotechnology Coccidiostats. https://www.ncbi.nlm.nih.gov/mesh/68003049.

[34] Drugbank Niclosamide. https://www.drugbank.ca/drugs/DB06803.

[35] Jaworska J., Nikolova-Jeliazkova N. (2007). How can structural similarity analysis help in category formation?. SAR QSAR Environ. Res..

[36] Torrent-Sucarrat M., De Proft F., Ayers P.W., Geerlings P. (2010). On the applicability of local softness and hardness. Phys. Chem. Chem. Phys..

[37] Vandewaterbeemd H., Kansy M. (1992). Hydrogen-bonding capacity and brain penetration. CHIMIA Int. J. Chem..

[38] Putz M.V., Ionaşcu C., Putz A.M., Ostafe V. (2011). Alert-QSAR. Implications for electrophilic theory of chemical carcinogenesis. Int. J. Mol. Sci..

[39] Oliferenko A.A., Krylenko P.V., Palyulin V.A., Zefirov N.S. (2002). A new scheme for electronegativity equalization as a source of electronic descriptors: Application to chemical reactivity. SAR QSAR Environ. Res..

[40] Hajimahdi Z., Safizadeh F., Zarghi A. (2016). Qsar analysis for some 1, 2-benzisothiazol-3-one derivatives as caspase-3 inhibitors by stepwise mlr method. Iran. J. Pharm. Res..

[41] Gozalbes R., Doucet J.P., Derouin F. (2002). Application of topological descriptions in QSAR and drug design: History and new trends. Curr. Drug Targets Infect. Disord..

[42] OECD Quantitative Structure-Activity Relationships Project [(Q)SARs] OECD. Principles for the Validation, for Regulatory Purposes of (Quantitative) Structure Activity Relationship Models. http://www.oecd.org/env/ehs/oecdquantitativestructure-activityrelationshipsprojectqsars.htm.

[43] Kar S.S., Bhat V.G., Shenoy V.P., Bairy I., Shenoy G.G. (2019). Design, synthesis, and evaluation of novel diphenyl ether derivatives against drug-susceptible and drug-resistant strains of Mycobacterium tuberculosis. Chem. Biol. Drug Des..

[44] Schulz D., Nachtigall J., Geisen U., Kalthoff H., Imhoff J.F., Fiedler H.P., Süssmuth R.D. (2012). Silvalactam, a 24-membered macrolactam antibiotic produced by Streptomyces sp. Tudie; 6392. J. Antibiot..

[45] Amagai K., Kudo F., Eguchi T. (2011). Biosynthetic pathway of macrolactam polyketide antibiotic cremimycin. Tetrahedron.

[46] Foss M.H., Pou S., Davidson P.M., Dunaj J.L., Winter R.W., Pou S., Licon M.H., Doh J.K., Li Y., Kelly J.X. (2016). Diphenylether-Modified 1,2-Diamines with Improved Drug Properties for Development against Mycobacterium tuberculosis. ACS Infect. Dis..

[47] Fothergill A.W. (2006). Miconazole: A historical perspective. Expert Rev. Anti. Infect. Ther..

[48] Vandenbosch D., Braeckmans K., Nelis H.J., Coenye T. (2010). Fungicidal activity of miconazole against Candida spp. biofilms. J. Antimicrob. Chemother..

[49] Mao K., Zhang X., Ali E., Liao X., Jin R., Ren Z., Wan H., Li J. (2019). Characterization of nitenpyram resistance in Nilaparvata lugens (Stål). Pestic. Biochem. Physiol..

[50] Hess C., Brockmann C., Doberentz E., Madea B., Musshoff F. (2014). Unintentional lethal overdose with metildigoxin in a 36-week-old infant—Post mortem tissue distribution of metildigoxin and its metabolites by liquid chromatography tandem mass spectrometry. Forensic Sci. Int..

[51] Dasgupta A. (2019). Issues of Interferences in Therapeutic Drug Monitoring. Biotin and Other Interferences in Immunoassays.

[52] Hauptman P.J., Kelly R.A. (1999). Digitalis. Circulation.

[53] Drugbank 2′,4′-Dinitrophenyl-2deoxy-2-Fluro-B-D-Cellobioside (CB04086). https://www.drugbank.ca/drugs/DB04086.

[54] González De La Huebra M.J., Vincent U., Bordin G., Rodríguez A.R. (2004). Characterisation of dirithromycin and spiramycin using high performance liquid chromatography and direct infusion mass spectrometry. Anal. Chim. Acta.

[55] Castaldo R.S., Celli B.R., Gomez F., LaVallee N., Souhrada J., Hanrahan J.P. (2003). A comparison of 5-day courses of dirithromycin and azithromycin in the treatment of acute exacerbations of chronic obstructive pulmonary disease. Clin. Ther..

[56] Wasilewski M.M., Wilson M.G., Slides G.D., Stotka J.L. (2000). Comparative efficacy of 5 days of dirithromycin and 7 days of erythromycin in skin and soft tissue infections. J. Antimicrob. Chemother..

[57] Chen T.F., Chang Y.C., Hsiao Y., Lee K.H., Hsiao Y.C., Lin Y.H., Tu Y.C.E., Huang H.C., Chen C.Y., Juan H.F. (2021). DockCoV2: A drug database against SARS-CoV-2. Nucleic Acids Res..

[58] Murugan N.A., Kumar S., Jeyakanthan J., Srivastava V. (2020). Searching for target-specific and multi-targeting organics for Covid-19 in the Drugbank database with a double scoring approach. Sci. Rep..

[59] Chowdhury K.H., Chowdhury M.R., Mahmud S., Tareq A.M., Hanif N.B., Banu N., Ali Reza A.S.M., Emran T.B., Simal-Gandara J. (2021). Drug repurposing approach against novel coronavirus disease (COVID-19) through virtual screening targeting SARS-CoV-2 main protease. Biology.

[60] Pantsar T., Poso A. (2018). Binding affinity via docking: Fact and fiction. Molecules.

[61] Warren G.L., Andrews C.W., Capelli A.M., Clarke B., LaLonde J., Lambert M.H., Lindvall M., Nevins N., Semus S.F., Senger S. (2006). A critical assessment of docking programs and scoring functions. J. Med. Chem..

[62] Grover M., Singh B., Bakshi M., Singh S. (2000). Quantitative structure-property relationships in pharmaceutical research—Part 1. Pharm. Sci. Technol. Today.

[63] Malhotra R., Ravesh A., Singh V. (2017). Synthesis, characterization, antimicrobial activities, and QSAR studies of organotin(IV) complexes. Phosphorus Sulfur Silicon Relat. Elem..

[64] Kumer A., Paul S. (2019). The Simulating Study of Homo, Lumo, Thermo Physical and Quantitative Structure of Activity Relationship (QSAR) of some Anticancer Active Ionic Liquids. Eurasian J. Environ. Res..

[65] Kumar A., Grewal A.S., Singh V., Narang R., Pandita D., Lather V. (2018). Synthesis, Antimicrobial Activity and QSAR Studies of Some New Sparfloxacin Derivatives. Pharm. Chem. J..

[66] Khodair A.I., Awad M.K., Gesson J.P., Elshaier Y.A.M.M. (2020). New N-ribosides and N-mannosides of rhodanine derivatives with anticancer activity on leukemia cell line: Design, synthesis, DFT and molecular modelling studies. Carbohydr. Res..

[67] Suresh Kumar S., Athimoolam S., Sridhar B. (2018). Structural, spectral, theoretical and anticancer studies on new co-crystal of the drug 5-fluorouracil. J. Mol. Struct..

[68] Kanagamani K., Muthukrishnan P., Ilayaraja M., Shankar K., Kathiresan A. (2018). Synthesis, Characterisation and DFT Studies of Stigmasterol Mediated Silver Nanoparticles and Their Anticancer Activity. J. Inorg. Organomet. Polym. Mater..

[69] Jeyaseelan S.C., Premkumar R., Kaviyarasu K., Franklin Benial A.M. (2019). Spectroscopic, quantum chemical, molecular docking and in vitro anticancer activity studies on 5-Methoxyindole-3-carboxaldehyde. J. Mol. Struct..

[70] Sarkar I., Goswami S., Majumder P. (2020). Quantitative structure–activity relationship (QSAR) study of some DNA-intercalating anticancer drugs. Computational Advancement in Communication Circuits and Systems.

[71] Wang J., Yun D., Yao J., Fu W., Huang F., Chen L., Wei T., Yu C., Xu H., Zhou X. (2018). Design, synthesis and QSAR study of novel isatin analogues inspired Michael acceptor as potential anticancer compounds. Eur. J. Med. Chem..

[72] Baeten A., Tafazoli M., Kirsch-Volders M., Geerlings P. (1999). Use of the HSAB principle in quantitative structure–activity relationships in toxicological research: Application to the genotoxicity of chlorinated hydrocarbons. Int. J. Quantum Chem..

[73] Bradbury S.P., Mekenyan O.G., Ankley G.T. (1998). The role of ligand flexibility in predicting biological activity: Structure-activity relationships for aryl hydrocarbon, estrogen, and androgen receptor binding affinity. Environ. Toxicol. Chem..

[74] Joshi R., Pandey N., Yadav S.K., Tilak R., Mishra H., Pokharia S. (2018). Synthesis, spectroscopic characterization, DFT studies and antifungal activity of (E)-4-amino-5-[*N*’-(2-nitro-benzylidene)-hydrazino]-2,4-dihydro-[1,2,4]triazole-3-thione. J. Mol. Struct..

[75] Joshi R., Kumari A., Singh K., Mishra H., Pokharia S. (2020). Triorganotin(IV) complexes of Schiff base derived from 1,2,4-triazole moiety: Synthesis, spectroscopic investigation, DFT studies, antifungal activity and molecular docking studies. J. Mol. Struct..

[76] Yan Z., Liu A., Huang M., Liu M., Pei H., Huang L., Yi H., Liu W., Hu A. (2018). Design, synthesis, DFT study and antifungal activity of the derivatives of pyrazolecarboxamide containing thiazole or oxazole ring. Eur. J. Med. Chem..

[77] Ali M.S., Farah M.A., Al-Lohedan H.A., Al-Anazi K.M. (2018). Comprehensive exploration of the anticancer activities of procaine and its binding with calf thymus DNA: A multi spectroscopic and molecular modelling study. RSC Adv..

[78] Rachedi K.O., Ouk T.S., Bahadi R., Bouzina A., Djouad S.E., Bechlem K., Zerrouki R., Ben Hadda T., Almalki F., Berredjem M. (2019). Synthesis, DFT and POM analyses of cytotoxicity activity of α-amidophosphonates derivatives: Identification of potential antiviral O,O-pharmacophore site. J. Mol. Struct..

[79] Da Costa R.M., Bastos J.K., Costa M.C.A., Ferreira M.M.C., Mizuno C.S., Caramori G.F., Nagurniak G.R., Simão M.R., dos Santos R.A., Veneziani R.C.S. (2018). In vitro cytotoxicity and structure-activity relationship approaches of ent-kaurenoic acid derivatives against human breast carcinoma cell line. Phytochemistry.

[80] Soffers A.E.M.F., Boersma M.G., Vaes W.H.J., Vervoort J., Tyrakowska B., Hermens J.L.M., Rietjens I.M.C.M. (2001). Computer-modeling-based QSARs for analyzing experimental data on biotransformation and toxicity. Toxicology In Vitro.

[81] Flores M.C., Márquez E.A., Mora J.R. (2018). Molecular modeling studies of bromopyrrole alkaloids as potential antimalarial compounds: A DFT approach. Med. Chem. Res..

[82] Cortes E., Mora J.R., Márquez E. (2020). Modelling the Anti-Methicillin-Resistant Staphylococcus Aureus (MRSA) Activity of Cannabinoids: A QSAR and Docking Study. Crystals.

[83] Mizukami Y. (2020). Character of Frontier Orbitals of Antiviral Drugs: Candidate Drugs against Covid-19. Open J. Phys. Chem..

[84] Hagar M., Ahmed H.A., Aljohani G., Alhaddad O.A. (2020). Investigation of some antiviral *N*-heterocycles as COVID 19 drug: Molecular docking and DFT calculations. Int. J. Mol. Sci..

[85] Valdés-Martiní J.R., Marrero-Ponce Y., García-Jacas C.R., Martinez-Mayorga K., Barigye S.J., D’Almeida Y.S.V., Pham-The H., Pérez-Giménez F., Morell C.A. (2017). QuBiLS-MAS, open source multi-platform software for atom- and bond-based topological (2D) and chiral (2.5D) algebraic molecular descriptors computations. J. Cheminform..

[86] Leonard J.T., Roy K. (2006). On selection of training and test sets for the development of predictive QSAR models. QSAR Comb. Sci..

[87] García-Jacas C.R., Marrero-Ponce Y., Acevedo-Martínez L., Barigye S.J., Valdés-Martiní J.R., Contreras-Torres E. (2014). QuBiLS-MIDAS: A parallel free-software for molecular descriptors computation based on multilinear algebraic maps. J. Comput. Chem..

[88] García-Jacas C.R., Marrero-Ponce Y., Cortés-Guzmán F., Suárez-Lezcano J., Martinez-Rios F.O., García-González L.A., Pupo-Meriño M., Martinez-Mayorga K. (2019). Enhancing Acute Oral Toxicity Predictions by using Consensus Modeling and Algebraic Form-Based 0D-to-2D Molecular Encodes. Chem. Res. Toxicol..

[89] Mora J.R., Marrero-Ponce Y., García-Jacas C.R., Suarez Causado A. (2020). Ensemble Models Based on QuBiLS-MAS Features and Shallow Learning for the Prediction of Drug-Induced Liver Toxicity: Improving Deep Learning and Traditional Approaches. Chem. Res. Toxicol..

[90] Marrero-Ponce Y., Iyarreta-Veitía M., Montero-Torres A., Romero-Zaldivar C., Brandt C.A., Ávila P.E., Kirchgatter K., Machado Y. (2005). Ligand-based virtual screening and in silico design of new antimalarial compounds using nonstochastic and stochastic total and atom-type quadratic maps. J. Chem. Inf. Model..

[91] Cabrera N., Mora J.R., Marquez E.A. (2019). Computational Molecular Modeling of Pin1 Inhibition Activity of Quinazoline, Benzophenone, and Pyrimidine Derivatives. J. Chem..

[92] Mora J.R., Márquez E.A., Calle L. (2018). Computational molecular modelling of N-cinnamoyl and hydroxycinnamoyl amides as potential α-glucosidase inhibitors. Med. Chem. Res..

[93] Márquez E., Mora J.R., Flores-Morales V., Insuasty D., Calle L. (2020). Modeling the antileukemia activity of ellipticine-related compounds: QSAR and molecular docking study. Molecules.

[94] Edraki N., Das U., Hemateenejad B., Dimmock J.R., Miri R. (2016). Comparative QSAR analysis of 3,5-bis (arylidene)-4-piperidone derivatives: The development of predictive cytotoxicity models. Iran. J. Pharm. Res..

[95] (2014). Matlab.

[96] Gramatica P. (2020). Principles of QSAR Modeling: Comments and Suggestions from Personal Experience. Int. J. Quant. Struct. Relationships.

[97] Schneidman-Duhovny D., Dror O., Inbar Y., Nussinov R., Wolfson H.J. (2008). Deterministic pharmacophore detection via multiple flexible alignment of drug-like molecules. J. Comput. Biol..

[98] Schneidman-Duhovny D., Dror O., Inbar Y., Nussinov R., Wolfson H.J. (2008). PharmaGist: A webserver for ligand-based pharmacophore detection. Nucleic Acids Res..

[99] Sunseri J., Koes D.R. (2016). Pharmit: Interactive exploration of chemical space. Nucleic Acids Res..

[100] Berman H.M., Battistuz T., Bhat T.N., Bluhm W.F., Bourne P.E., Burkhardt K., Feng Z., Gilliland G.L., Iype L., Jain S. (2002). The protein data bank. Acta Crystallogr. Sect. D Biol. Crystallogr..

[101] Jin Z., Du X., Xu Y., Deng Y., Liu M., Zhao Y., Zhang B., Li X., Zhang L., Peng C. (2020). Structure of Mpro from SARS-CoV-2 and discovery of its inhibitors. Nature.

[102] (2015). The PyMol Molecular Graphics System.

[103] Morris G.M., Huey R., Lindstrom W., Sanner M.F., Belew R.K., Goodsell D.S., Olson A.J. (2010). AutoDock4 and AutoDockTools4: Automated Docking with Selective Receptor Flexibility. J. Comput. Chem..

[104] Trott O., Olson A.J. (2010). AutoDock Vina: Improving the Speed and Accuracy of Docking with a New Scoring Function, Efficient Optimization, and Multithreading. J. Comput. Chem..

[105] Laskowski R.A., Swindells M.B. (2011). LigPlot+: Multiple ligand-protein interaction diagrams for drug discovery. J. Chem. Inf. Model..

[106] (2020). Discovery Studio Visualizer.

[107] Van Der Spoel D., Lindahl E., Hess B., Groenhof G., Mark A.E., Berendsen H.J.C. (2005). GROMACS: Fast, flexible, and free. J. Comput. Chem..

[108] Vanommeslaeghe K., Hatcher E., Acharya C., Kundu S., Zhong S., Shim J., Darian E., Guvench O., Lopes P., Vorobyov I. (2010). CHARMM general force field: A force field for drug-like molecules compatible with the CHARMM all-atom additive biological force fields. J. Comput. Chem..

[109] Berendsen H.J.C., Postma J.P.M., Van Gunsteren W.F., Dinola A., Haak J.R. (1984). Molecular dynamics with coupling to an external bath. J. Chem. Phys..

[110] Kumari R., Kumar R., Consortium O.S.D.D., Lynn A. (2014). g_mmpbsa—A GROMACS tool for MM-PBSA and its optimization for high-throughput binding energy calculations. J. Chem. Inf. Model..

[111] Adolfo Cuesta S., Cordova-Sintjago T., Ramón Mora J. (2020). Sulfonylation of Five-Membered Aromatic Heterocycles Compounds through Nucleophilic Aromatic Substitution: Concerted or Stepwise Mechanism?. ChemistrySelect.

[112] Cervantes C., Mora J.R., Marquez E., Torres J., Rincón L., Mendez M.A., Alcázar J.J. (2019). Theoretical calculations of the multistep reaction mechanism involved in asparagine pyrolysis supported by degree of rate control and thermodynamic control analyses. Appl. Sci..

[113] Frisch M.J.G., Trucks W., Schlegel H.B., Scuseria G.E., Robb M.A., Cheeseman J.R., Scalmani G., Barone V., Mennucci B., Petersson G.A. (2016). Gaussian 16.

