# Supplementary material for: In Silico Screening of the DrugBank Database to Search for Possible Drugs against SARS-CoV-2
Source: Molecules. 2021 Feb 19;26(4):1100. doi: 10.3390/molecules26041100 (PMC7923184; doi:10.3390/molecules26041100)
Supplement: Supplementary file 1 [file molecules-26-01100-s001.zip › supporting information/SM3.pdf]

In silico screening of the DrugBank Database to Search for Possible Drugs against SARS-CoV-2.

Sebastián A. Cuesta [1], José R. Mora [1,*], Edgar A. Márquez[2]

[1] Universidad San Francisco de Quito, Grupo de Química Computacional y Teórica (QCT-USFQ), Departamento de Ingeniería Química, Diego de Robles y Vía Interoceánica, Quito 170901, Ecuador
[2] Grupo de Investigaciones en Química y Biología, Departamento de Química y Biología, Facultad de Ciencias Exactas, Universidad del Norte, Carrera 51B, Km 5, vía Puerto Colombia, Barranquilla 081007, Colombia

Supplemental material

Table S1. Models obtained for the 2D, 3D, and combined modeling

	**Model**	**N° of attributes**	**Descriptors**
2D modelling	M1	7	SD_B_AB_Ci(2.0;-2.0)_2_SS1_H_n_X_NSRW_e-h_MAS<br>N3_B_BB_nCi_2_SS2_H_T_KA_c-p_MAS<br>N2_Q_AB_nCi_2_MP0_H_T_KA_a_MAS MN_Q_AB_nCi_2_SS5_H_n_X_LGP[2-<br>4]_c_MAS K_B_AB_Ci(2.0;-2.0)_2_NS8_H_n_X_LGP[4-6]_e-c_MAS<br>K_B_AB_nCi_2_NS7_T_LGP[2-4]_r-c_MAS S_F_AB_nCi_2_NS2_X_LGP[2-<br>4]_a_MAS
	M2	6	SD_B_AB_Ci(2.0;-2.0)_2_SS1_H_n_X_NSRW_e-h_MAS<br>N3_B_BB_nCi_2_SS2_H_T_KA_c-p_MAS MN_Q_AB_nCi_2_SS5_H_n_X_LGP[2-<br>4]_c_MAS K_B_AB_Ci(2.0;-2.0)_2_NS8_H_n_X_LGP[4-6]_e-c_MAS<br>K_B_AB_nCi_2_NS7_T_LGP[2-4]_r-c_MAS S_F_AB_nCi_2_NS2_X_LGP[2-<br>4]_a_MAS
	M3	5	N3_B_BB_nCi_2_SS2_H_T_KA_c-p_MAS MN_Q_AB_nCi_2_SS5_H_n_X_LGP[2-<br>4]_c_MAS K_B_AB_Ci(2.0;-2.0)_2_NS8_H_n_X_LGP[4-6]_e-c_MAS<br>K_B_AB_nCi_2_NS7_T_LGP[2-4]_r-c_MAS S_F_AB_nCi_2_NS2_X_LGP[2-<br>4]_a_MAS
	M4	4	N3_B_BB_nCi_2_SS2_H_T_KA_c-p_MAS MN_Q_AB_nCi_2_SS5_H_n_X_LGP[2-<br>4]_c_MAS K_B_AB_nCi_2_NS7_T_LGP[2-4]_r-c_MAS<br>S_F_AB_nCi_2_NS2_X_LGP[2-4]_a_MAS
3D modelling	M5	7	ES_SD_B_AB_nCi_2_M12_SS2_T_LGP[5]_psa-p_MID<br>AC[3]_S_Q_AB_nCi_2_M14_MP7_T_LGL[1-2]_h_MID<br>AC[4]_S_B_AB_nCi_2_M3_SS6_o_T_LGP[2]_m-h_MID<br>IB_S_F_AB_nCi_2_M8_SS6_o_T_LGL[5-6]_c_MID<br>SD_B_AB_nCi_2_M10_SS2_A_LGL[5-6]_a-p_MID<br>AC[1]_K_B_AB_nCi_2_M5_NS6_T_LGL[1-2]_h-s_MID<br>Q2_F_AB_nCi_2_M8_NS1_o_T_LGP[2]_e_MID
	M6	6	AC[3]_S_Q_AB_nCi_2_M14_MP7_T_LGL[1-2]_h_MID<br>AC[4]_S_B_AB_nCi_2_M3_SS6_o_T_LGP[2]_m-h_MID<br>IB_S_F_AB_nCi_2_M8_SS6_o_T_LGL[5-6]_c_MID<br>SD_B_AB_nCi_2_M10_SS2_A_LGL[5-6]_a-p_MID<br>AC[1]_K_B_AB_nCi_2_M5_NS6_T_LGL[1-2]_h-s_MID<br>Q2_F_AB_nCi_2_M8_NS1_o_T_LGP[2]_e_MID
	M7	5	AC[4]_S_B_AB_nCi_2_M3_SS6_o_T_LGP[2]_m-h_MID<br>IB_S_F_AB_nCi_2_M8_SS6_o_T_LGL[5-6]_c_MID<br>SD_B_AB_nCi_2_M10_SS2_A_LGL[5-6]_a-p_MID<br>AC[1]_K_B_AB_nCi_2_M5_NS6_T_LGL[1-2]_h-s_MID<br>Q2_F_AB_nCi_2_M8_NS1_o_T_LGP[2]_e_MID
	M8	4	IB_S_F_AB_nCi_2_M8_SS6_o_T_LGL[5-6]_c_MID<br>SD_B_AB_nCi_2_M10_SS2_A_LGL[5-6]_a-p_MID<br>AC[1]_K_B_AB_nCi_2_M5_NS6_T_LGL[1-2]_h-s_MID<br>Q2_F_AB_nCi_2_M8_NS1_o_T_LGP[2]_e_MID
2D-3D modelling (combined)	M9	7	N3_Q_AB_nCi_2_SS3_n_T_KA_c_MAS<br>N1_B_AB_nCi_2_M11_MP4_T_LGP[1]_v-e_MID<br>IB_K_B_AB_nCi_2_M8_NS6_T_LGP[2]_a-p_MID<br>IB_S_F_AB_nCi_2_M8_SS6_o_T_LGL[5-6]_c_MID

			SD_B_AB_nCi_2_M10_SS2_A_LGL[5-6]_a-p_MID<br>AC[1]_K_B_AB_nCi_2_M5_NS6_T_LGL[1-2]_h-s_MID<br>AC[1]_S_F_AB_nCi_2_M14_MP1_n_T_LGP[5]_a_MID			
	M10	6	N3_Q_AB_nCi_2_SS3_n_T_KA_c_MAS<br>N1_B_AB_nCi_2_M11_MP4_T_LGP[1]_v-e_MID<br>IB_K_B_AB_nCi_2_M8_NS6_T_LGP[2]_a-p_MID<br>IB_S_F_AB_nCi_2_M8_SS6_o_T_LGL[5-6]_c_MID<br>SD_B_AB_nCi_2_M10_SS2_A_LGL[5-6]_a-p_MID<br>AC[1]_K_B_AB_nCi_2_M5_NS6_T_LGL[1-2]_h-s_MID			
	M11	5	N3_Q_AB_nCi_2_SS3_n_T_KA_c_MAS<br>N1_B_AB_nCi_2_M11_MP4_T_LGP[1]_v-e_MID<br>IB_S_F_AB_nCi_2_M8_SS6_o_T_LGL[5-6]_c_MID<br>SD_B_AB_nCi_2_M10_SS2_A_LGL[5-6]_a-p_MID<br>AC[1]_K_B_AB_nCi_2_M5_NS6_T_LGL[1-2]_h-s_MID			
	M12	4	N3_Q_AB_nCi_2_SS3_n_T_KA_c_MAS<br>IB_K_B_AB_nCi_2_M8_NS6_T_LGP[2]_a-p_MID<br>SD_B_AB_nCi_2_M10_SS2_A_LGL[5-6]_a-p_MID<br>AC[1]_K_B_AB_nCi_2_M5_NS6_T_LGL[1-2]_h-s_MID			
	Model	R^2	Q^2_{LOO}	Q^2_{boot}	Q^2_{ext}	a(R2)
2D modelling	M1	0.901	0.831	0.776	0.924	0.147
	M2	0.878	0.781	0.741	0.935	0.114
	M3	0.841	0.731	0.703	0.887	0.093
	M4	0.815	0.716	0.686	0.873	0.055
3D modelling	M5	0.912	0.860	0.822	0.931	0.153
	M6	0.878	0.810	0.770	0.892	0.137
	M7	0.841	0.783	0.741	0.752	0.090
	M8	0.792	0.731	0.697	0.625	0.058
2D-3D modelling (combined)	M9	0.935	0.889	0.862	0.945	0.148
	M10	0.901	0.851	0.815	0.879	0.124
	M11	0.871	0.813	0.776	0.782	0.086
	M12	0.824	0.771	0.732	0.836	0.055
	Model	a(Q2)	Kx	Kxy	F	s
2D modelling	M1	-0.518	0.305	0.384	35.04	0.163
	M2	-0.392	0.310	0.396	33.57	0.177
	M3	-0.328	0.253	0.359	30.57	0.199
	M4	-0.285	0.198	0.327	33.01	0.211
3D modelling	M5	-0.441	0.297	0.376	39.83	0.153
	M6	-0.353	0.271	0.376	33.58	0.177
	M7	-0.326	0.221	0.352	30.57	0.199
	M8	-0.268	0.150	0.329	28.57	0.223
2D-3D modelling (combined)	M9	-0.440	0.295	0.379	55.66	0.131
	M10	-0.372	0.252	0.354	42.47	0.160
	M11	-0.322	0.202	0.332	39.13	0.179
	M12	-0.277	0.278	0.339	34.99	0.206

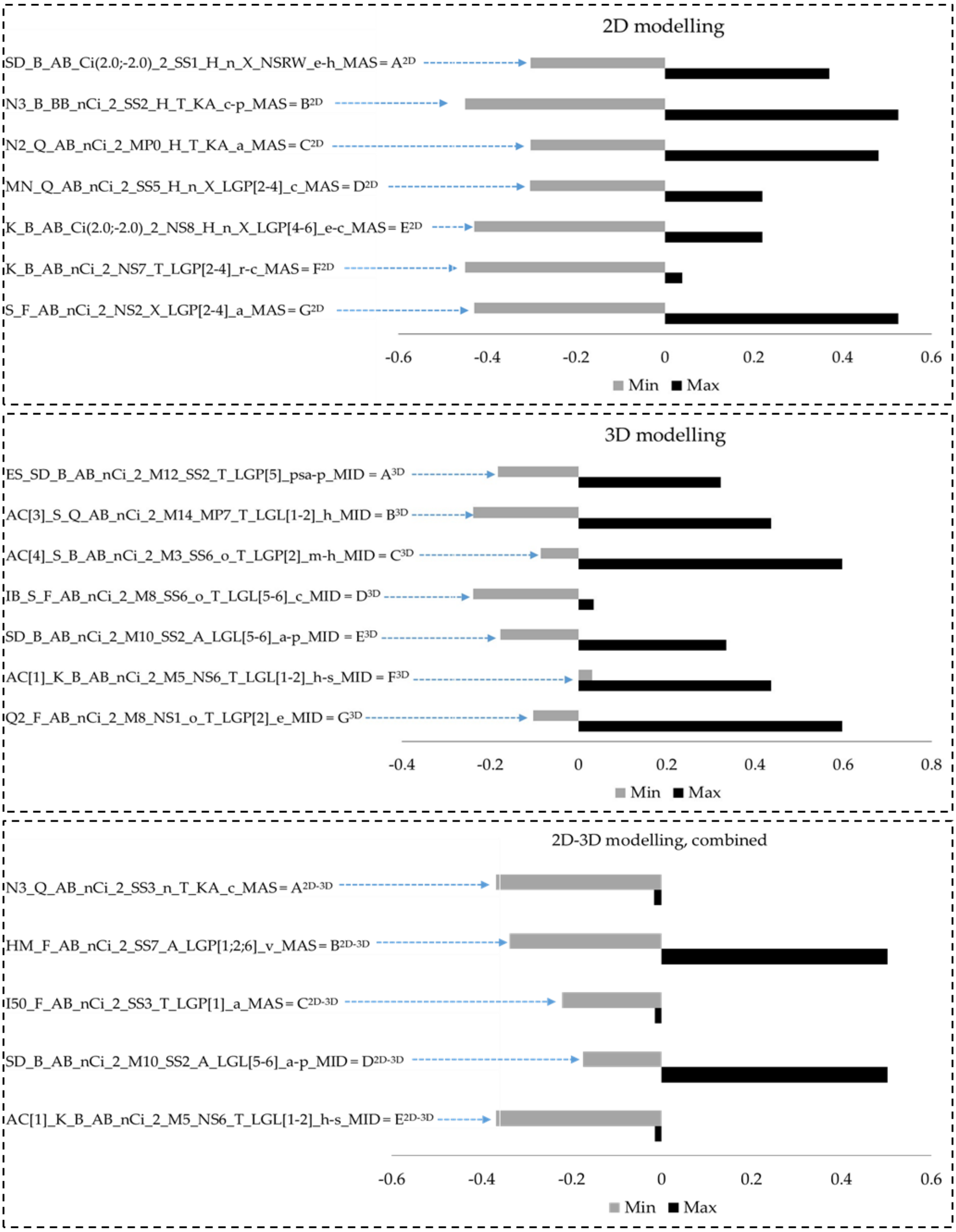

Figure S1. Pearson's coefficient range for each descriptor concerning the other descriptors

Table S2. DockCov2 scores of compounds in Jeon et.al Database

Drug name	pIC_{50}	DockCov2 score			
		Highest score	Target	Mpro	RdRp
Digoxin	6.72	-11.2	ACE2	-9.1	-8.8
Digitoxin	6.64	-12.19	Npro	-8.8	-10.2
Salinomycin. sodium	6.62	--	--	--	--
Niclosamide	6.55	-8.3	Npro	-7.1	-7.5
Hexachlorophene	6.05	-7.7	Npro	-6.7	-6.9
Proscillaridin	5.69	-10.2	Npro	-8.6	-8.6
LDK378	5.54	-9.8	Npro	-7.9	-7.5
Tetrandrine	5.52	--	--	--	--
Isoosajin	5.49	--	--	--	--
Osimertinib mesylate	5.49	-3.9	RdRp	-3.6	-3.9
Bazedoxifene	5.46	-9.4	Npro	-8.1	-7.2
Toremifene citrate	5.45	-7.4	Npro	-6.3	-7.2
Oxyclozanide	5.43	--	--	--	--
Lusutrombopag	5.42	--	--	--	--
Dronedarone HCl	5.41	-7.8	Npro	-6.4	-6.2
Osajin	5.41	-8	Npro	-6.7	-6.7
Tilorone	5.39	--	--	--	--
Ciclesonide	5.36	-9.5	ACE2	-8.2	-8.6
Mefloquine	5.36	-9	Npro	-7.6	-7.8
Cepharanthine	5.35	--	--	--	--
Isopomiferin	5.35	--	--	--	--
Anidulafungin	5.33				
Penfluridol	5.3	-9.7	ACE2	-8.1	-9.1
Amodiaquine dihydrochloride	5.29	--	--	--	--
Clomiphene citrate	5.27	-6.5	TMPRSS2	-5.1	-5.7
Cyclosporine	5.24	-8	ACE2	-6.7	-7.6
Hydroxyprogesterone caproate	5.2	-9.1	Npro	-6.7	-7.2
Perhexiline maleate	5.2	--	--	--	--
Abemaciclib	5.18	-10.1	Npro	-8.3	-8.5
Droloxifene	5.18	--	--	--	--
Ivacaftor	5.18	-9.8	Npro	-7.1	-8.3
Gilteritinib	5.17	--	--	--	--
Thioridazine hydrochloride	5.17	-7.9	Npro	-6.6	-7.1
Ebastine	5.16	-9.3	ACE2	-6.6	-8.3
Triparanol	5.15	--	--	--	--
Chloroquine diphosphate	5.14	-4.4	RdRp	-4.1	-4.4
Mequitazine	5.14	-7.8	Npro	-6.7	-7
Berbamine hydrochloride	5.1				
Eltrombopag	5.08	-10.7	Npro	-8.1	-9.7
Lopinavir	5.04	-9.7	Npro	-8.7	-9.3
Loperamide hydrochloride	5.03	-9.3	Npro	-7.6	-8.4
Remdesivir	4.94	--	--	--	--

Dihydrogambogic_acid	4.87	--	--	--	--
Phenazopyridine hydrochloride	4.55	-6.8	Npro	-5.8	-6.5

Nucleocapsid (N) protein (Npro), angiotensin-converting enzyme 2 (ACE2), transmembrane serine protease family member II (TMPRSS2).

Table S3. Jeon et. Al. Database screening using pharmacophore models 4 and 5

Drug name	pIC50	RMSD	
		Model 4	Model 5
Digoxin	6.72	0.10697	0.24221
Digitoxin	6.64	0.25856	0.19464
Salinomycin, sodium	6.62	0.34615	0.00011
Niclosamide	6.55	--	--
Hexachlorophene	6.05	--	--
Proscillaridin	5.69	0.07881	--
LDK378	5.54	--	--
Tetrandrine	5.52	0.54294	--
Osimertinib mesylate	5.49	--	0.21731
Isoosajin	5.49	--	--
Bazedoxifene	5.46	--	--
Toremifene citrate	5.45	--	--
Oxyclozanide	5.43	--	--
Lusutrombopag	5.42	0.24496	--
Dronedarone HCl	5.41	0.27583	--
Osajin	5.41	--	--
Tilorone	5.39	0.41212	0.31106
Ciclesonide	5.36	0.31142	0.32088
Mefloquine	5.36	--	--
Isopomiferin	5.35	0.30104	--
Cepharanthine	5.35	--	--
Anidulafungin	5.33	0.13258	0.12907
Penfluridol	5.3	--	--
Amodiaquine dihydrochloride	5.29	--	--
Clomiphene citrate	5.27	--	--
Cyclosporine	5.24	--	0.12426
Hydroxyprogesterone caproate	5.2	--	--
Perhexiline maleate	5.2	--	--
Abemaciclib	5.18	--	--
Droloxifene	5.18	--	--
Ivacaftor	5.18	--	--
Gilteritinib	5.17	--	0.25692
Thioridazine hydrochloride	5.17	--	--
Ebastine	5.16	--	--
Triparanol	5.15	0.29577	--
Chloroquine diphosphate	5.14		--
Mequitazine	5.14		--
Berbamine hydrochloride	5.1		0.15365
Eltrombopag	5.08	0.30189	0.26311
Lopinavir	5.04	--	0.14669

Loperamide hydrochloride	5.03	--	--
Remdesivir	4.94	0.21888	0.27564
Dihydrogambogic_acid	4.87	0.41089	0.07281
Phenazopyridine hydrochloride	4.55	--	--

Table S4. Best compounds for the DrugBank screening using pharmacophore models 4 and 5

**Model 4**			**Model 5**		
**DrugBank ID**	**pIC_{50}***	**RMSD**	**DrugBank ID**	**pIC_{50}***	**RMSD**
DB03259	6,9	0,27	DB02438	6,9	0,04
DB01980	6,7	0,28	DB02213	6,8	0,08
DB08956	7,1	0,35	DB01980	6,7	0,09
DB04652	6,9	0,48	DB03259	6,9	0,10
DB02438	6,9	0,52	DB08943	6,6	0,21
DB00486	6,9	0,53	DB03024	6,7	0,29
			DB01481	6,8	0,30

**Predicted Values*

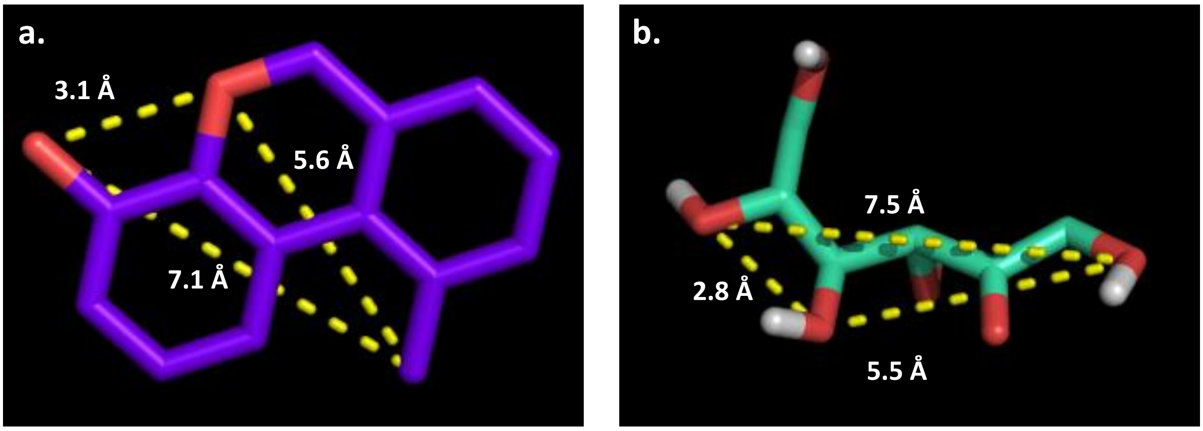

Figure S2. a. Pharmacophore model 4 fitting DB03259. b. Pharmacophore model 5 fitting DB02438.

Table S5. Docking results of the Dataset against Mpro, PLpro, and RdRp, ordered from largest to lowest pIC_{50}

		**Docking Score (kcal/mol)**				
**Drug name**	**PubChem ID**	**Mpro**	**PLpro**	**RdRp**	**pIC_{50}**	**Dataset**
Digoxin	2724385	-9.2	-8.5	-9.4	6.72	Training
Digitoxin	441207	-7.8	-8.9	-9.3	6.64	Test
Salinomycin, sodium	23682228	-8.0	-7.2	-8.6	6.62	Training
Niclosamide	4477	-7.0	-6.8	-6.3	6.55	Training
Hexachlorophene	3598	-5.9	-6.8	-6.0	6.05	Training
Proscillaridin	5284613	-7.8	-7.5	-8.4	5.69	Training
LDK378	57379345	-8.1	-7.1	-8.5	5.54	Training

Tetrandrine	73078	-7.2	-7.5	-9.1	5.52	Training
Isoosajin	1069873	-8.9	-7.5	-8.2	5.49	Training
Osimertinib mesylate	78357807	-7.1	-6.9	-7.3	5.49	Training
Bazedoxifene	154257	-7.8	-7.1	-7.8	5.46	Training
Toremifene citrate	3005572	-6.5	-6.2	-6.2	5.45	Test
Oxyclozanide	16779	-6.6	-6.6	-6.5	5.43	Training
Lusutrombopag	49843517	-7.1	-7.2	-7.3	5.42	Training
Dronedarone HCl	219025	-6.2	-5.9	-5.8	5.41	Test
Osajin	95168	-7.9	-7.2	-7.4	5.41	Training
Tilorone	5475	-6.1	-5.4	-6.1	5.39	Training
Ciclesonide	6918155	-8.2	-7.7	-8.3	5.36	Training
Mefloquine	4046	-8.0	-7.0	-7.0	5.36	Training
Cepharanthine	10206	-8.8	-7.7	-9.0	5.35	Training
Isopomiferin	20055152	-8.8	-7.7	-8.4	5.35	Training
Anidulafungin	166548	-4.7	-6.7	-9.3	5.33	Training
Penfluridol	33630	-7.9	-6.9	-8.4	5.30	Test
Amodiaquine dihydrochloride	64646	-7.2	-6.0	-6.1	5.29	Training
Clomiphene citrate	3033832	-5.8	-6.0	-6.9	5.27	Training
Cyclosporine	5284373	-5.8	-6.0	-7.3	5.24	Test
Hydroxyprogesterone caproate	169870	-6.8	-6.1	-6.5	5.20	Training
Perhexiline maleate	5284439	-6.5	-6.1	-5.7	5.20	Training
Abemaciclib	46220502	-8.3	-7.5	-8.1	5.18	Test
Droloxifene	3033767	-6.4	-6.7	-6.5	5.18	Test
Ivacaftor	16220172	-7.3	-6.8	-7.3	5.18	Training
Gilteritinib	49803313	-8.3	-7.5	-7.9	5.17	Training
Thioridazine hydrochloride	66062	-7.2	-6.9	-6.8	5.17	Test
Ebastine	3191	-7.5	-6.7	-7.0	5.16	Test
Triparanol	6536	-6.9	-6.2	-6.6	5.15	Training
Chloroquine diphosphate	64927	-5.9	-5.5	-5.3	5.14	Training
Mequitazine	4066	-7.0	-6.9	-6.6	5.14	Training
Berbamine hydrochloride	56845155	-7.6	-7.4	-8.5	5.10	Training
Eltrombopag	135449332	-8.6	-8.8	-8.8	5.08	Training
Lopinavir	92727	-7.0	-6.2	-6.9	5.04	Training
Loperamide hydrochloride	71420	-7.1	-7.1	-7.6	5.03	Training
Remdesivir	121304016	-8.1	-6.3	-7.3	4.94	Training
Dihydrogambogic_acid	6857793	-8.3	-7.3	-8.7	4.87	Training
Phenazopyridine hydrochloride	8691	-6.3	-5.7	-6.3	4.55	Training

Table S6. Docking results of the Best Molecules of the DrugBank screening, ordered from largest to loweest predicted pIC_{50}

		Docking Score (kcal/mol)			
**Drug name**	**DrugBank ID**	**Mpro**	**PLpro**	**RdRp**	**pIC_{50}***
Para-iodo-D-phenylalanine hydroxamic acid	DB01980	-5.7	-5.9	-5.8	6.67
2',6'-Dichloro-Biphenyl-2,6-Diol	DB03259	-6.3	-6.2	-6.0	6.86
3-O-Methylfructose	DB02438	-4.8	-4.7	-4.5	6.90
Metanitrophenyl-Alpha-D-Galactoside	DB02213	-6.7	-6.1	-7.8	6.77
3-amino-azacyclotridecan-2-one	DB08476	-5.9	-5.7	-6.1	7.51
2-(2,4-dichlorophenoxy)-5-(pyridin-2-ylmethyl)phenol	DB07287	-6.6	-6.6	-7.3	7.50

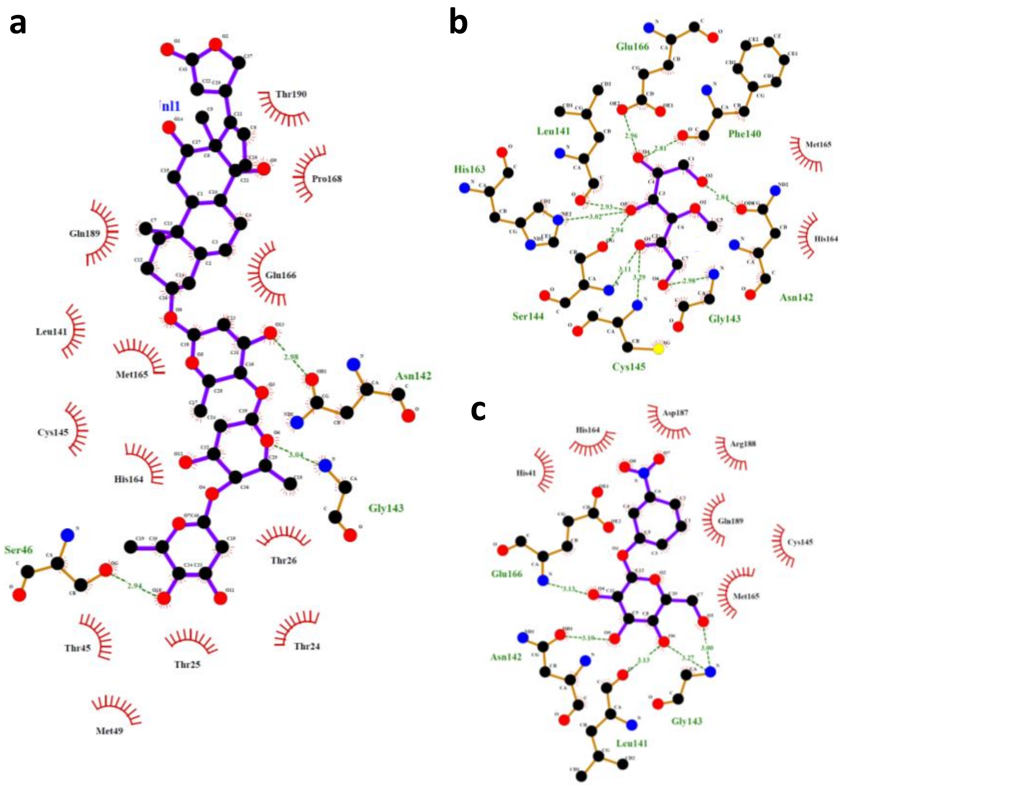

Figure S3. 2D representation of the interaction of Digoxin (a), DB02438 (b), and DB02213 (c) with the active site of Mpro.

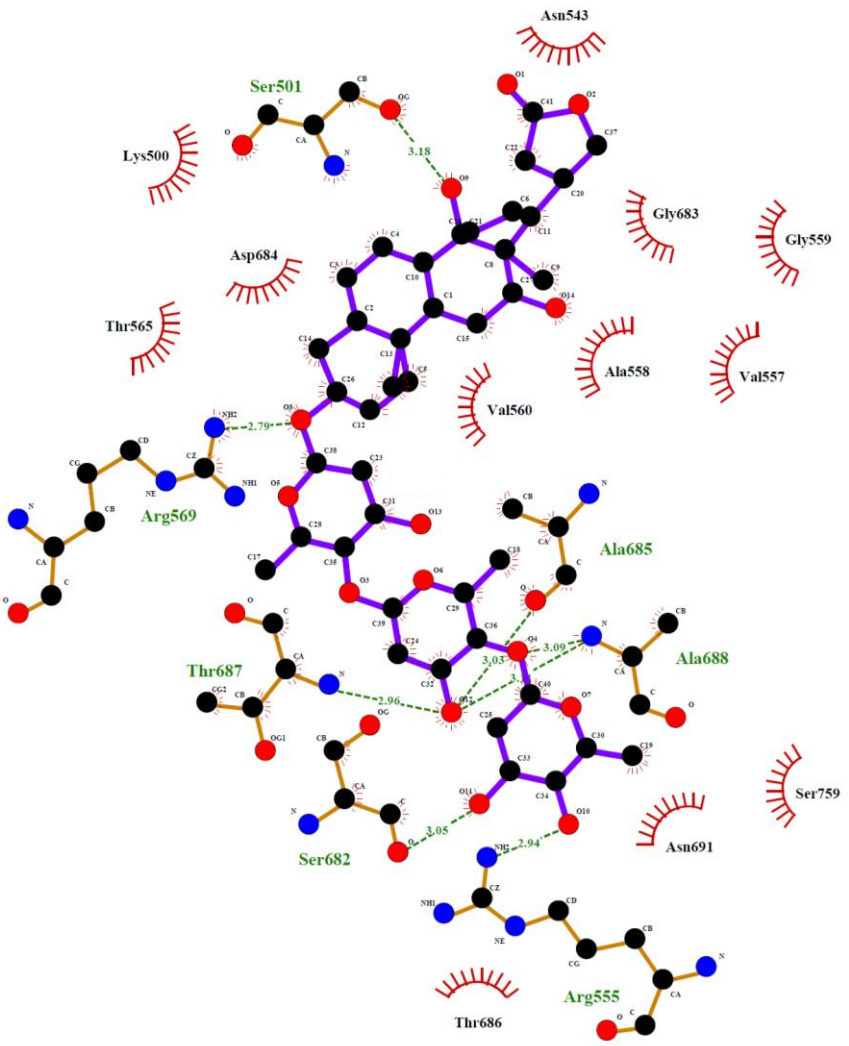

Figure S4. 2D representation of the interaction of Digoxin with the active site of RdRp

.

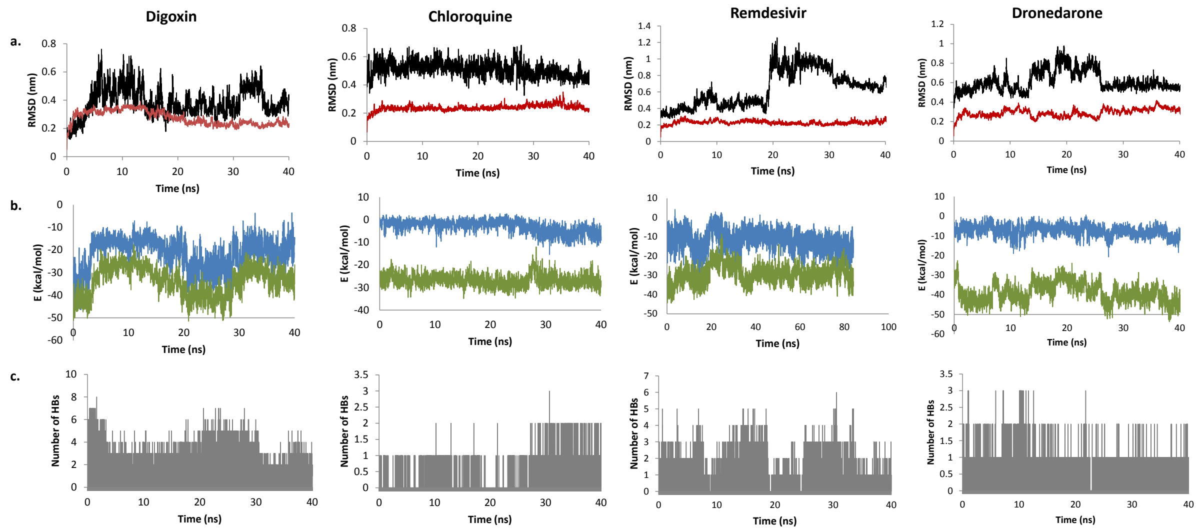

Figure S5. MD results for Mpro. a. RMSD of the protein-ligand complex (red) and the ligand (black). b. Coulomb (blue) and Lennard-Jones (green) interaction energy. c. the number of hydrogen bonds.

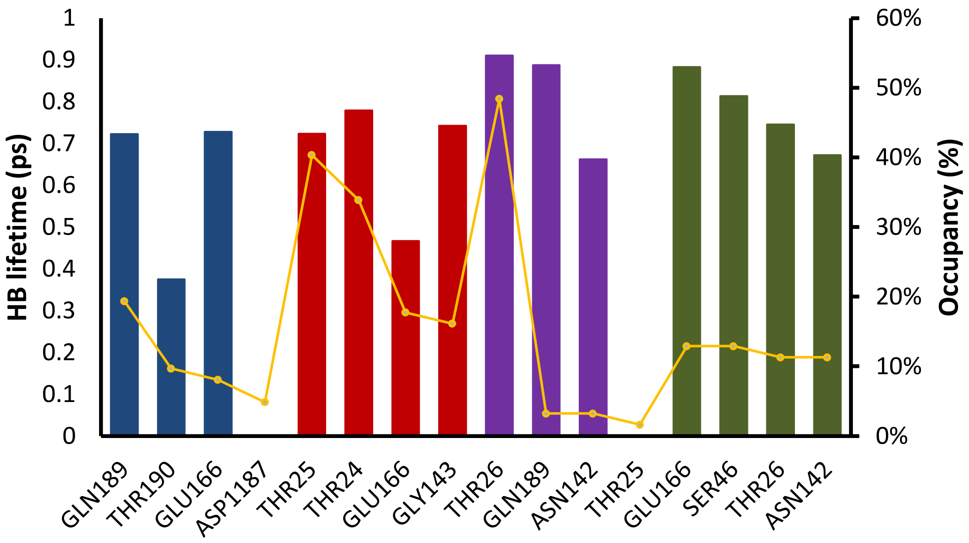

Figure S6. Occupancy (yellow line) and HB lifetimes (Bars) for the MD results of Mpro. Chloroquine in blue, digoxin in red, dronedarone in purple, and remdesivir in green.

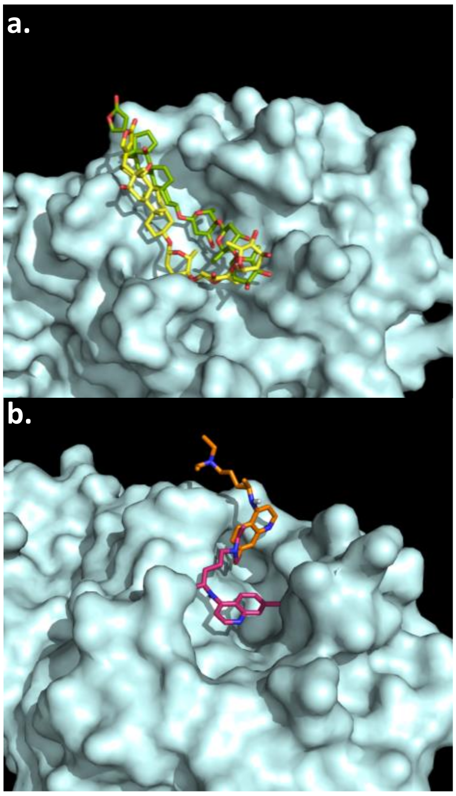

Figure S7. Interaction of MPro (pale cyan) with a. Docking (yellow) and MD (green) results for Digoxin. b. Docking (pink) and MD (orange) result for Chloroquine.

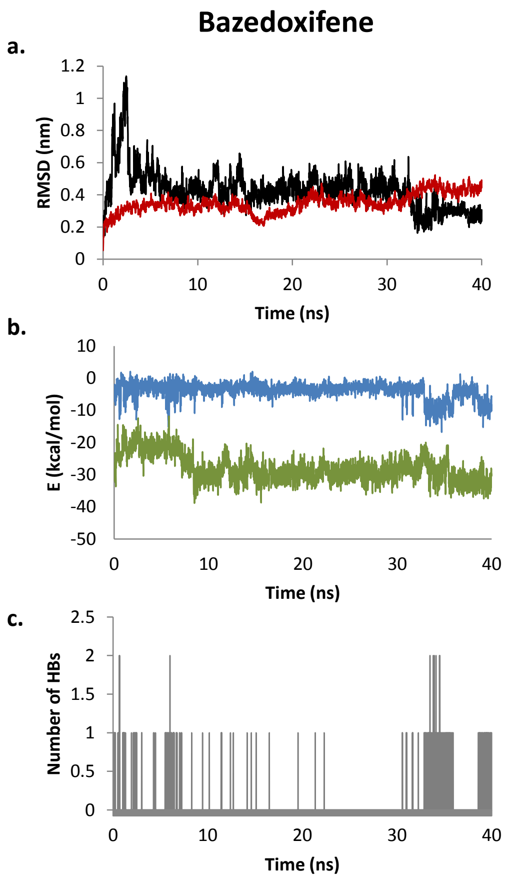

Figure S8. MD results for PLpro interaction with bazedoxifene. a. RMSD of the protein-ligand complex (red) and the ligand (black). b. Coulomb (blue) and Lennard-Jones (green) interaction energy. c. the number of hydrogen bonds.

Table S7. Gris box used for the docking study

Parameter	Mpro	PLpro	RdRp
X	-10.712	22.007	113.641
Y	12.411	69.530	117.604
Z	68.831	2.961	130.823
Size X	20	30	32
Size Y	24	30	32
Size Z	20	30	32
